# Is a correlation-based investment strategy beneficial for long-term international portfolio investors?

**DOI:** 10.1186/s40854-023-00471-9

**Published:** 2023-03-09

**Authors:** Seema Wati Narayan, Mobeen Ur Rehman, Yi-Shuai Ren, Chaoqun Ma

**Affiliations:** 1 Asia Pacific Applied Economics Association, Melbourne, Australia; 2 Shaheed Zulfikar Ali Bhutto Institute of Science and Technology (SZABIST), Islamabad, Pakistan; 3 South Ural State University, 76 Lenin Prospekt, Chelyabinsk, Russian Federation; 4 School of Public Administration, Hunan University, Changsha, China; 5 Research Institute of Digital Society and Blockchain, Hunan University, Changsha, China; 6 Centre for Resource and Environmental Management, Hunan University, Changsha, China; 7 The Energy Centre, University of Auckland, Auckland, New Zealand; 8 Business School, Hunan University, Changsha, China

**Keywords:** Portfolio diversification, Portfolio mix, Asia, Central and Eastern Europe, Middle East North Africa, Latin America, G11, G15, F3, F65

## Abstract

Using negative to low-correlated assets to manage short-term portfolio risk is not uncommon among investors, although the long-term benefits of this strategy remain unclear. This study examines the long-term benefits of the correlation strategy for portfolios based on the stock market in Asia, Central and Eastern Europe, the Middle East and North Africa, and Latin America from 2000 to 2016. Our strategy is as follows. We develop five portfolios based on the average unconditional correlation between domestic and foreign assets from 2000 to 2016. This yields five regional portfolios based on low to high correlations. In the presence of selected economic and financial conditions, long-term diversification gains for each regional portfolio are evaluated using a panel cointegration-based testing method. Consistent across all portfolios and regions, our key cointegration results suggest that selecting a low-correlated portfolio to maximize diversification gains does not necessarily result in long-term diversification gains. Our empirical method, which also permits the estimation of cointegrating regressions, provides the opportunity to evaluate the impact of oil prices, U.S. stock market fluctuations, and investor sentiments on regional portfolios, as well as to hedge against these fluctuations. Finally, we extend our data to cover the years 2017–2022 and find that our main findings are robust.

**Supplementary Information:**

The online version contains supplementary material available at 10.1186/s40854-023-00471-9.

## Introduction

In recent years, numerous academics and research institutions have concentrated on portfolio rebalancing to mitigate portfolio risks. Although some studies have addressed whether diversifying international portfolios into emerging market countries benefits investors, most studies have not yet accounted for the investment strategies employed, and the benefits for long-term investors remain unknown. Stock market volatility will significantly impact capital market and economic growth (Wen et al. 2019). Understanding institutionally sensible and long-term productive investment policies is thus critical for a country’s development.

The portfolio’s goal, as the core of investment strategies, is to regulate its systematic risk to the market and protect the entire portfolio’s security and income. Portfolio theory postulates that higher diversification gains can be obtained if the investment portfolio is composed of weakly correlated assets. Combining an asset with a negative or low correlation with other assets in a portfolio result in superior risk-adjusted returns (Markowitz 1952). This approach is readily used to diversify risks over the lifetime of a portfolio investment, although its long-term benefits are not well understood, and financial data distribution is typically very complex (Li et al. 2021).

Given the importance of weakly correlated assets in portfolios and to the best of our knowledge, there is no research on the impact of weakly correlated asset portfolios on the long-term benefits of investors. Therefore, to elucidate the long-term diversification benefits of such a strategy, this study develops correlation-based portfolios for emerging stock markets in Asia, Central and Eastern Europe (CEE), the Middle East and North Africa (MENA), and Latin America, taking into account a commonly used rebalancing/hedging strategy. This study specifically considers a long-term investor from an emerging nation (*i*) who invests in the domestic market (*i*) and all other emerging markets (*js*) in Asia, CEE, Latin America, and MENA. Such an investor also rebalances or hedges his or her portfolio based on the unconditional correlations between *i* and *js*. Rather than grouping all *j* markets into a single portfolio, we develop five portfolios based on the unconditional correlation between markets (*i*) and (*j*) over the study period. We then use cointegration methods and vector error correction models (VECMs) to test long-term comovement between (*i)* and (*j)* emerging markets for each region. To assess the long-term relationships under different economic/financial conditions, we condition the relationships between (*i*) and (*js*) with the S&P 500, oil price, global investor sentiment, and S&P 500 during the Global Financial Crisis (GFC) and non-GFC (NGFC) period.

The empirical models used to evaluate the five portfolios are similar to the autoregressive conditional heteroskedasticity (ARCH) or generalized ARCH (GARCH) family, which is commonly used to evaluate the spillover effects of international markets on domestic markets. However, we use panel cointegration and VECM estimation methods because we are interested in the long-term benefits of the correlation-based portfolio development strategy. These methods can test whether or not a cointegrating or stable long-term equilibrium relationship exists between *i* and *j* equity markets. Furthermore, cointegration can be easily used to assess long-term diversification gains; that is, if markets are cointegrated, on the same long-term equilibrium path(s), or comove in the long-term, diversification gains are predicted to be weak, and vice versa.

A strand of the portfolio diversification literature assesses long-term diversification gains using the cointegration method. However, three key features distinguish the current study from previous research. First, the current study is the first to examine long-term diversification gains for portfolios of emerging market Morgan Stanley Capital International (MSCI) organized by pairwise unconditional correlations. Previous studies did not necessarily consider any portfolio development strategy, whereas the current study examines portfolios developed using a rebalancing and hedging strategy. As Narayan and Rehman (2021) pointed out, most previous cointegration-based studies on equity markets considered regionally biased strategies because they examined at the pairwise cointegration relationship between markets in the same region.

To develop the correlation-based portfolios, we first calculate the unconditional correlations between an emerging stock market MSCI (*i*)) against the rest of the emerging stock markets MSCIs (*j*) in the four regions over the study period (2000–2016) (Additional file 1: Table S1). Here, emerging market (*i*) refers to a domestic emerging nation, whereas emerging market (*j*) refers to an emerging foreign country. This is repeated for all nations in the four regions. Moreover, although Markowitz’s (1952) portfolio theory suggests that a low-correlated portfolio provides the best opportunities for portfolio diversification, we develop and evaluate portfolios with low-correlated to high-correlated assets to ensure completeness in our analyses.

Hence, for each emerging stock market (*i*), we divide the (*j*) emerging markets into five portfolios (yellow, blue, green, purple, and red) based on the size of the correlations between *i* and *js* (Additional file 1: Table S2). MSCIs (*js*) are negatively to lowly correlated, are divided into yellow and blue portfolios for each region. The green portfolio includes the medium correlated emerging market MSCIs, whereas the purple and red portfolios include the highly correlated emerging market MSCIs. Next, region-specific panels are developed, with five panels for each region. Each panel includes stock markets from all countries in a region and a portfolio of other emerging markets from all four regions. We consider this panel setup to be reliable because regression of the *i*’s against each of the five *j* portfolios by region produces the logical effect one would expect from this setup—that highly correlated (*j*) markets have a greater influence on domestic markets (*i*) than less correlated (*j*) markets.

Second, as previously stated, the present study examines a portfolio of MSCIs from all four regions for a country’s representative investor. Modeling multiple assets simultaneously caters for a more realistic scenario in which investors diversify domestic equity market risk with more than one international asset. However, the literature is only concerned with pairwise relationships, for example, in Asia (Candelon et al. 2008; Gupta and Guidi 2012; Batareddy et al. 2012), CEE (Linne 1998; Demian 2011; Caporale and Spagnolo 2012; Bieńkowski et al. 2014), MENA (Gündüz and Omran 2001; Yu and Hassan 2008; Chortareas et al. 2011), and Latin America (Choudry 1997; Chen et al. 2002). These studies are explained in Sect. "Literature review".

Third, in this study, the cointegration relationship between domestic markets and each of the five portfolios (with low-to-high-correlated assets) is evaluated in the presence of some economic and financial economic conditions, namely the U.S. stock market, Brent oil market, investor sentiments, and the GFC. However, the literature focuses on the spillover effects of other equity markets on the domestic market without considering such shocks. With the rapid development and diversification of the economy and finance (Kou et al. 2021), some studies take U.S. stock market shocks into account when examining a cointegration relationship between equity markets (Diamandis 2009; Abid et al. 2014; Lin and Lin 2018). A few recent studies evaluating portfolio diversification gains within Asia emphasize the importance of accounting for economic and financial conditions (Narayan and Rehman 2017, 2018, 2020).

To foreshadow our key results, the cointegration results, which are consistent across all portfolios and regions, suggest that selecting a low-correlated portfolio to maximize diversification gains does not always translate into long-term diversification gains. We also find that investors in the four regions can improve their long-term diversification opportunities by hedging oil prices and changes in the U.S. stock market. Our findings imply that, in times of higher oil prices and negative S&P 500 shocks, emerging market investors can reduce their long-term vulnerability to these shocks by including (or switching from) minimally to highly correlated emerging markets in their portfolio. Lowly correlated emerging markets in the portfolio are beneficial because they have the least impact and act as effects to protect the domestic emerging market from varying investor sentiment.

Given that our primary research uses average correlations calculated between 2000 and 2016 to develop the various portfolios, this suggests that investors have less-than-perfect knowledge when constructing their portfolios. We conduct the following to account for even more flaws in our knowledge. Using the correlation strategy applied over 2000–2016 (as shown in Additional file 1: Table S2), we re-develop portfolios over the recent years (2017–2022), replicating the behavior of investors who used prior knowledge (in this case, on the correlation strategy) to develop their portfolio over 2017–2022. We then reassess the long-term benefits of the newly developed portfolios. Although many portfolios constructed in this manner are found to be unsuitable for the long-term analysis performed here, this method that allows for imperfect knowledge confirms that our main findings are robust.

The remainder of the paper is structured as follows.  "Literature review" section reviews the literature. "Empirical method" section describes the empirical model and the estimation method. Data and preliminary data analysis" section describes the data and provides preliminary analyses of the data. "Empirical results" section presents the empirical results. "Further analysis" section delves into our out-of-sample analysis and key findings. Finally, "Conclusion" section concludes the paper.

## Literature review

We present studies that test for the long-term diversification opportunities between national equity markets in Asia, CEE, MENA, and Latin America. According to the literature, a portfolio should include two or a few regionally biased national equity markets. It looks for pairwise cointegrating relationships (mostly in time series) without considering other portfolio rebalancing or hedging strategies. Several studies consider developed markets in their cointegration tests, whereas others consider economic and financial conditions. The following are brief explanations of key features and findings from this literature by region.

In Asia, studies offer mixed findings on long-term diversification gains (Narayan and Rehman 2020). Gupta and Guidi (2012) found no evidence of a stable long-term relationship between India and three Asian markets (i.e., Hong Kong, Japan, or Singapore) over a decade (1999–2009), implying potential benefits to investors looking to expand their portfolio with these markets. Meanwhile, Batareddy et al. (2012) found a similar result in the emerging stock markets of India, China, South Korea, and Taiwan. Studies before 2010 did find evidence of pairwise cointegration, including Manning (2002) for nine Asian markets from 1988 to 1999, and Mukherjee and Bose (2008) for the Indian stock market and major Asian stock markets from January 1999 to June 2005. Candelon et al. (2008) found this in five East Asian countries during the Asian financial crisis in 1997, but they interpreted it as a sign of contagion. Using panel cointegration methods, Narayan and Rehman (2017, 2018) showed evidence of cointegrating relationships between selected Asian markets from 2000 to 2013 at daily, weekly, and monthly data frequencies, suggesting limited daily, weekly, and monthly diversification opportunities within Asia. However, Narayan and Rehman (2020) found limited evidence of cointegration by applying the same methodology separately for frontier or emerging Asian markets over the extended period to 2018. Even after controlling for some economic and financial factors such as oil prices and the GFC, Narayan and Rehman (2021) found no evidence of cointegration within Asia over the period 2000–2016. Although the authors confirmed that an investor can profit differently from emerging or frontier Asian markets and both markets combined, they also clarified that diversification opportunities with Asian markets have changed rapidly since the GFC. Only a few studies have used more recent data, including data from the GFC, to find evidence of cointegration or increased comovement between Asian markets (Narayan and Rehman 2017, 2018, 2020, 2021). The present paper examines the effects of the GFC and other economic and financial conditions in Asia.

Most researchers have studied long-term comovement or a cointegrating relationship between CEE markets in light of the GFC and other economic and financial conditions. Linne (1998) found a lack of evidence of a cointegrating link between developed and CEE markets during 1991–1997, leading the author to conclude that CEE markets are primarily driven by their peculiarities. However, in a study over the period 2001–2009, Demian (2011) found increased evidence of cointegration between CEE markets since E.U. accession, although the author contends that economic and financial factors were more important instigators of this increased comovement. Bieńkowski et al. (2014) examined long-term stock market comovement in three CEE countries (Poland, the Czech Republic, and Hungary) over the period 2005–2013, including the GFC (2007–2009) and the Euro-area sovereign debt crisis (2010–2013). They used daily data to find a vector autoregressive-GARCH (VAR-GARCH) model and discovered that the stock markets of the CEE-3 economies only became slightly more integrated after joining the European Union. The GFC was found to positively impact the correlation between the CEE-3 markets. Meanwhile, the Euro-area sovereign debt crisis weakened the stock market correlations and negatively impacted stock market indices’ returns. Nonetheless, the impact of the sovereign debt crisis was less intense and profound than the impact of the GFC. Although the rate of return transmission played a role, volatility spillovers were more important. Caporale and Spagnolo (2012) developed a VAR-GARCH model for the same three CEE nations from 1999 to 2005 and found stronger links since the E.U. accession.

Cointegration studies on the MENA and Latin American markets are limited. For instance, Gündüz and Omran (2001) found no evidence of a stable cointegrating relationship between MENA stock markets. Chortareas et al. (2011) investigated the relationship between stock markets, exchange rates, and oil prices in four MENA countries (Egypt, Kuwait, Oman, and Saudi Arabia) over 1994–2006 and found no evidence of a stable long-term relationship between the variables for the four MENA nations. Meanwhile, Yu and Hassan (2008) demonstrated a cointegrating relationship between the Gulf Cooperation Council (GCC) and non-GCC MENA nations from 1999 to 2005. Choudry (1997) found evidence of cointegration between six Latin American markets. Chen et al. (2002) examined the interdependence of Latin American nations from 1995 to 2000 using cointegration and VECM methods. The authors find strong evidence of cointegration prior to and following the Asian and Russian financial crises.

Several studies have examined long- and short-term linkages between stock markets within the four regions, with developed markets, primarily the U.S., included in their portfolios (Diamandis 2009; Abid et al. 2014; Narayan et al. 2014; Lin and Lin 2018; Narayan and Rehman 2018, 2020). Abid et al. (2014) used the intertemporal capital asset pricing model framework to examine the dynamics of regional integration among South Asian markets. The authors found that risk is regionally priced and that the U.S. term premium and market are the primary drivers of regional integration. Using a time-varying regression model of returns, Lin and Lin (2018) examined the short-term impact of the U.S. and China as leading players on selected Asian nations. They found that the U.S. exerts more influence on Asia than China, after adding control variables, such as the global MSCI index, the Asian financial crisis, and the GFC. Lin and Lin (2018) found an increase in China’s influence on Asian markets while highlighting the U.S.’s declining influence, particularly after the GFC. After examining time-varying dynamic conditional correlations for selected Asian markets against the U.S. and China, Narayan et al. (2014) reached a similar conclusion. Meanwhile, Narayan and Rehman (2018) found that the U.S. stock market was a significant predictor of emerging and frontier Asian (EFA) markets over the period 2000–2013. They found that although the Forex and oil markets reduce the importance of developed stock markets, they are not as important to EFA markets as the U.S. market. Narayan and Rehman (2020) showed differences in long- and short-term gains between the U.S. and emerging and frontier Asian markets due to differences in data frequencies and investment end dates.

Some studies, such as Dooley and Hutchison (2009), found a stronger correlation between the U.S. and other emerging markets during the GFC period than during the NGFC period. Narayan and Rehman (2017) used daily, weekly, and monthly data frequencies to examine the link between selected Asian stock markets and the Dow Jones Industrial Average (DJIA) from 2000 to 2013. The authors manipulated the DJIA using two binary variables representing the GFC and NGFC periods. The study found that the DJIA and Asian markets were significantly correlated during the GFC period, but not during the NGFC period. The authors suggested that monthly average data cannot show such effects, implying that the effect of the GFC on the correlation between the Asian markets and the DJIA was only temporary. Narayan and Rehman (2020) examined the link between the frontier and emerging Asian markets compared with developed markets under the same conditions and found that previous results hold true.

According to Chlibi et al. (2016), there was no stable long-term link between the U.S. and MENA (divided into GCC and non-GCC nations) before or after the GFC. Meanwhile, Röckinger and Ugra (2001) examine four CEE equity markets to the German, UK, and U.S. stock markets from 1994 to 1997. Although they found evidence of cointegration against London and Frankfurt, no evidence of cointegration between the CEE and the U.S. was discovered. Similarly, Gilmore and McManus (2002) found no long-term cointegration between the CEE and U.S. markets from 1995 to 2001. Yu and Hassan (2008) showed a cointegrating relationship between non-GCC MENA nations and the U.S. stock market. Using weekly data over the period January 1998 to July 2006, Diamandis (2009) found evidence of partial integration between the U.S. stock market and the Latin American stock markets of Argentina, Brazil, Chile, and Mexico. The authors found only one cointegrating vector within the group but four common trends. They also found that, despite the group’s limited long-term benefits from portfolio diversification, stock price adjustment to the common tends to be slow.

## Empirical method

In line with the literature, five models are examined^1^:

1 $${P}_{i,r,t}={\delta }_{1}+ {\theta }_{1}{P}_{j, r-i,t}+{\mu }_{1t}$$

2 $${P}_{i,r,t}={\delta }_{2}+ {\theta }_{21}{P}_{j,r-i,t}+ {\theta }_{22}{S\&P500}_{t}+{\mu }_{2t}$$

3 $${P}_{i,r,t}={\delta }_{3}+ {\theta }_{31}{P}_{j,r-i,t}+ {\theta }_{32}{S\&P500}_{t}+{\theta }_{33}{Brent}_{t}+{\mu }_{3t}$$

4 $${P}_{i,r,t}={\delta }_{4}+ {\theta }_{41}{P}_{j,r-i,t}+ {\theta }_{42}{S\&P500}_{t}{+ \theta }_{43}{sentiments}_{t}+{\mu }_{4t}$$

5 $${P}_{i,r,t}={\delta }_{5}+ {\theta }_{51}{P}_{j,r-i,t}+ {\theta }_{52}GFC*{S\&P500}_{t}+{\theta }_{53}NGFC*{S\&P500}_{t}{+\mu }_{5t}$$

Here, the dependent variable, ${P}_{i,r,t}$ is a vector of MSCI-based price index corresponding to each nation, $i$, in the region, $r$. Our main regressor, ${P}_{j,r-i,t}$, is a vector of the MSCI of emerging countries excluding country, *i*. ${P}_{j,r-i,t}$ is the emerging market-based investment portfolio developed for investors in each nation (*i*) with five portfolios based on the unconditional correlation between ${\Delta P}_{i}$ and ${\Delta P}_{j}$.

The unconditional correlations between the markets (*i* and *j*) are presented in Additional file 1: Tables S1 and Table S2 specifies the low-to-high correlation-based portfolios developed for each emerging market. Under the correlations between $\Delta {P}_{i,r,t}$ and $\Delta {P}_{j,r-i,t},$ five different sets of ${P}_{j,r-i,t}$ are created for each region,$r$. Panel 1 (yellow portfolio) contains ${P}_{j,r-i,t}$ with a correlation of less than zero with${P}_{i,r,t}$. Panel 2 (blue portfolio) contains ${P}_{j,r-i,t}$ with a correlation of 0.1 or lower with ${P}_{i,r,t }.$ Panel 3 (purple portfolio) contains ${P}_{j,r-i,t}$ with 0.2–0.3 correlation with${P}_{i,r,t}$. Panel 4 (green portfolio) has a 0.4–0.5 correlation. Panel 5 (red portfolio) has a correlation of 0.6 or more. Hence in total, five panels are developed under *S1* for each region.

At this point, we should mention that this study aims to compare long-term equilibrium relationships and the resulting diversification gains for portfolios with low-to-high correlated assets. As a result, we divide assets into portfolios based on the sample unconditional correlations between domestic emerging market (*i*) and foreign emerging markets (*j*).

Our other regressors are as follows: ${S\&P500}_{t}$ is the benchmark price index of the U.S. market; ${Brent}_{t}$ is the international Brent oil price series; and ${sentiments}_{t}$ is the investor sentiment index from the Baker and Wurgler (2006) study. The effect of the S&P 500 is also captured during the GFC and NGFC periods in model (5). These two binary variables, GFC and NGFC, are developed following Dooley and Hutchison (2009). $\delta s$ and $\theta s$ are the parameters to be estimated.

Models (1)–(5) are estimated for five panels per region. Model (1) is the primary model. It examines the relationship between an emerging equity market and foreign markets. Models (2)–(5) are used for robustness testing. Models (2)–(5) examine the relationship between a domestic emerging stock market and foreign emerging markets*,* as conditioned by the U.S. market, oil price, investor sentiment, and the U.S. market during the *GFC* and *NGFC* periods, respectively. Because all stock price data is in U.S. dollars, the models must account for fluctuations in the exchange rate.

These models are examined using three cointegration tests: Kao (1999), the Johansen–Fisher test (Maddala and Wu 1999), and Pedroni (1999, 2004). The Kao and Pedroni tests are panel versions of the Engle and Granger (1987) residual-based cointegration test and test the null hypothesis of no cointegration. The Kao test assumes panel data homogeneity and takes into account regressors that are either endogenous or exogenous. To evaluate the long-term relationship between the dependent and independent variables, the Kao test estimates a least squares dummy variable model with variables integrated to order one, *I (1*). Kao (1999) used the Dickey–Fuller autoregressive model and the augmented Dickey–Fuller autoregressive model to test the residuals, ${\mu }_{n,t}$, where $n=1,..5$ in models (1)–(5). For the existence of a long-term relationship, ${\widehat{\mu }}_{\mathrm{n},\mathrm{t}}$ needs to be *I (0)*.

Pedroni (1999, 2004) developed the panel cointegration test for estimating fixed effects. Unlike the Kao test, Pedroni (1999, 2004) covered for homogeneity and heterogeneity panels. The fixed effect estimations yield 11 individual and group statistics, all asymptotically converting to the normal distribution (Pedroni 1999, 2004).

Maddala and Wu (1999) developed their panel cointegration test by combining the Fisher (1992) method and the Johansen (1988) cointegration test. Hence, although the Pedroni and Kao tests use a single equation, the Johansen cointegration test employs a system of equations. Consequently, the Fisher–Johansen test employs the trace test and the maximum eigenvalue test to determine the number of cointegration relations between system variables. The Fisher–Johansen test also accounts for endogenous and exogenous variables, making it more versatile than the Kao test. Overall, there is no better estimation method. All three are used because they address different aspects of our samples, such as the presence of heterogeneous panels and the endogeneity problem.

Moreover, we estimate the VECM using ordinary least squares (OLS) with White’s (1980) consistent covariance matrix. We report the equation of interest:

6 $$\Delta {Y}_{t}={\delta }_{2i}+{\theta }_{1i}\sum_{k=1}^{n}\Delta {Y}_{t-k}+{\theta }_{2i}\sum_{k=1}^{n}\Delta {x}_{1;t-k}+{\delta }_{1i}EC{T}_{t-1}+{\varepsilon }_{t}$$

All variables appear in the model in the first difference form, denoted by $\Delta$. $\delta ,$ $\theta$, and λ are parameters estimated; The *ECT* is the error correction term (ECT) extracted from the long-term model, namely models (1) – (5). The ECT represents the deviation of *Y* from its long-term equilibrium value. ${\delta }_{1i}$ measures the speed of adjustment back to the long-term equilibrium after a shock to ${Y}_{t}$. The negative and significance of ${\delta }_{1i}$ confirms that the long-term relationship between the* x* and* Y* variables are stable. We use the Akaike Information Criteria using the lag length, estimated by VAR model analysis.

## Data and preliminary data analysis

This study employs daily MSCI (U.S. dollar) indices of the emerging markets of Asia, CEE, MENA, and Latin America from January 3, 2000, to December 30, 2016.^2^ Control variables include the S&P 500, Brent oil price, and U.S. investor sentiment. DataStream is used to extract all equity price indices. Brent oil price, in U.S. dollars, is extracted from the Energy Information Administration database. Baker and Wurgler (2006) provided the U.S. investor sentiment index.

We begin the preliminary analysis with the return form of the ${P}_{i}$ series, which depicts the domestic MSCI price index. Additional file 1: Table S3 contains the common statistics on MSCI-based prices and returns from January 3, 2000, to December 30, 2016. Latin America has the highest average MSCI return, followed by Asia, CEE, and MENA. Most stock return series have negative skewness, and the kurtosis coefficient for all return series is greater than 3.

**Fig. 1:**
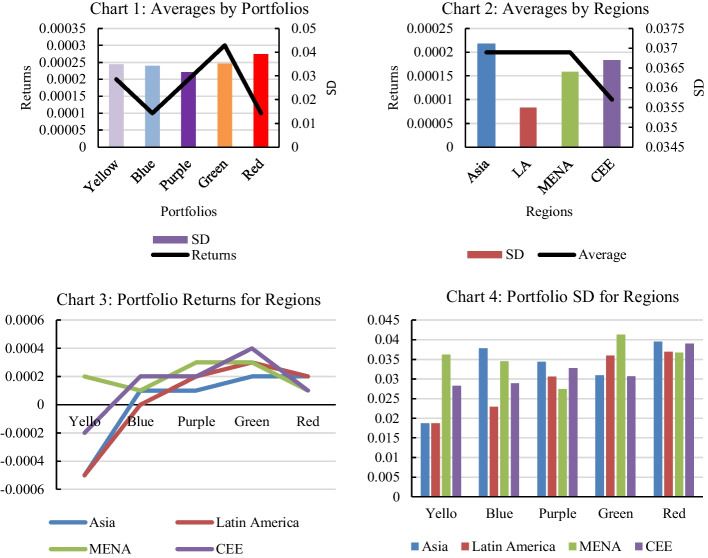
Average returns and standard deviation (SD) for portfolios and regions. *Note* This figure presents the average returns and risks individual nations under the high-low correlation strategy where equity portfolios for each country are chosen based on correlation with the rest of the emerging markets. Correlations between (*i*) and (*j*) markets are reported in Additional file 1: Table S1. Yellow portfolio captures correlation of less than zero; Blue portfolio captures correlation between zero to 0.1; Purple portfolio captures correlation between 0.2 and 0.3; Green portfolio covers correlation between 0.4 and 0.5; the Red portfolio stock consists of correlations of 0.6 or more. Individual country outcomes in Table 1 are used here to derive: (1) an average of each of the four portfolios (rows 3–6); and (2) an average of each of the four portfolios by regions

The return form of the ${P}_{j,r-i,t}$, which is the emerging market-based portfolio for each emerging nation, is developed using investment strategies based on high-to-low correlation. Figure 1 (Charts 1–4) compare the average return and risks associated with the five portfolios across the four regions. Interestingly, when we look at the average returns of the portfolios (with positive correlations), we see an inverted V-shape emerge as we move from the low-correlated portfolio (blue) to the highest-correlated portfolio (red) (Chart 1). In other words, the least (blue) and most (red) correlated emerging market portfolios have lower average returns, while those (purple and/or green) portfolios that fall between the blue and red portfolios have the highest average return. The yellow portfolio, which covers *j* markets that are least (or negatively) correlated with *i* markets, follows the aforementioned pattern on average, with lower returns than the blue or any other portfolio (Chart 3). However, in the case of MENA, the yellow portfolio outperforms the blue portfolio (Chart 3) in terms of average positive returns.

In terms of the risks associated with each portfolio, we note that the red portfolio, which contains the most correlated equities with the domestic stock market, has the highest standard deviation or risk (Chart 4). Except for MENA, which has the highest risk with the yellow portfolio, this is true for all.

The differences in average risk and return across the five portfolios and four regions suggest that these portfolios are worth investigating (Fig. 1). In the following empirical analysis, we examine at how these two investment strategies perform in the face of shocks in each region.

## Empirical results

This section uses cointegration and VECM methods to explain the long-term relationship between each emerging market region (*i*) and (*j*) portfolios with low-to-high correlated assets. Before conducting panel cointegration tests to examine the time series properties *i* and *j* portfolios, we performed the standard panel unit root tests, namely Levin et al. (2002) and Im et al. (2003). Non-tabulated results suggest that all return series are stationary, or *I (0*), and all price series are non-stationary, or *I (1)*. These results are available on request from the authors. Overall, our findings suggest that when it comes to establishing cointegration relationships, or short-term regression results, the different emerging market-based portfolios developed under the portfolios with high-to-low-correlated assets do not matter. However, portfolios with high- or low-correlated assets matter in the case of long-term regression analysis of cointegrated variables.

### Cointegration results for portfolios developed to maximize diversification gains

The panel cointegration tests, namely Kao (1999), the Johansen–Fisher test (Maddala and Wu (1999), and Pedroni (1999, 2004), are performed for all five (*j*) portfolios across the five models for the four emerging market groups, as explained above. Table 1 shows the results for the lowest correlated (or yellow) portfolio for Asia, CEE, MENA, and Latin America, whereas Table 2 shows a summary of all results. The detailed results for all four regions, are presented in the appendix in Additional file 1: Tables S4-1 to S4-4. We find that results vary significantly by region, but that model (3) produces a fairly robust finding of cointegration, or an equilibrium long-term, relationship between the variables considered, namely, ${P}_{i}$, ${P}_{j}$, $Brent$, and $S\&P 500$. These results indicate limited diversification gains in the long-term.

**Table 1:** Cointegration test results: yellow portfolio (with less than zero correlations)

	Kao panel cointegration	Pedroni panel co-integration statistics							Johansen panel co-integration trace statistics			
	ADF t-stat	Panel v	Panel rho	Panel PP	Panel ADF	Group rho	Group PP	Group ADF	None	1	2	3
*LA*												
Model 1	0.4160	− 0.6238	2.1795	2.6466	1.9126	1.0996	2.7184	2.0212	42.30*	43.33**	–	–
Model 2	0.3969	− 0.3749	2.5042	3.1265	2.4914	− 0.1579	1.5407	1.2916	32.23	5.569	18.74	–
Model 3	− 1.6241***	0.6371	− 6.6226***	− 2.2712***	− 5.1189***	− 16.9983***	− 7.4989***	− 8.1826***	185.3***	11.59	5.094	15.72
Model 4	0.3497	− 1.3825	3.6101	4.6507	4.1339	1.2067	2.6241	2.9529	305.7*	29.65*	5.868	19.56
Model 5	0.1736	− 1.0190	3.0244	4.0489	3.5063	1.3114	2.9784	2.6473	206.7	21.87	9.896	24.88
*Asia*												
Model 1	13.5631***	− 3.6780	6.6961	12.6149	11.3547	5.5517	8.6540	7.2703	32.12	49.66	–	–
Model 2	11.3911***	− 1.6565	4.0094	8.2164	2.4923	4.6895***	7.4666	4.1972***	25.23	16.30	41.87	–
Model 3	7.7204***	− 1.7432	− 14.5937***	− 5.3256***	− 0.8439	− 30.6208***	− 10.0924***	− 2.7905***	430.9	16.41	13.46	40.16
Model 4	11.3327***	− 2.7828	4.9511	9.3655	4.4256	6.1118	9.0771	6.6113	533.6***	25.23	16.29	44.16
Model 5	10.7290***	− 3.0391	6.0418	11.6179	6.6078	5.7789	9.2017	5.8151	289.6***	33.18	20.25	44.43
*MENA*												
Model 1	− 2.8796***	− 2.4877	1.3300	− 0.2854	0.6072	3.4566	0.9096	2.0721	57.91	72.02	–	–
Model 2	− 3.1270	− 3.3706	2.8525	1.4027	2.3832	5.2727	2.7841	3.9132	23.94	10.55	28.19	–
Model 3	− 4.7220***	− 3.5705	− 33.7749***	− 19.1868***	− 9.0839***	− 34.4190***	− 12.6639***	− 7.0891***	797.5***	19.21	9.861	27.34
Model 4	− 3.0597***	− 4.2968	4.0298	3.2197	4.7451	6.3704	4.8312	6.6672	727.6***	24.33	10.69	30.00
Model 5	− 2.4614***	− 4.1531	3.6532	3.1554	3.9341	6.5984	5.5017	6.3870	423.4***	43.04	20.30	45.52
*CEE*												
Model 1	2.1718***	− 1.8941	1.2917	1.2650	1.3205	4.3549	4.8544	5.2865	44.90	45.95	–	–
Model 2	1.6542***	0.1380	1.8323	1.7037	1.3980	2.9751	3.0016	3.1958	68.20***	14.21	50.91	–
Model 3	− 2.0754	2.5500***	− 88.3206***	− 34.8370***	− 20.7549***	− 13.2459***	− 2.5790***	− 1.3759***	358.2***	39.82	14.15	49.55
Model 4	1.6099**	− 1.1975	3.2900	3.3722	3.3318	2.2373	1.7243	4.9906	449.1***	66.14**	14.84	51.49
Model 5	1.6162***	− 0.5555	2.1435	1.9935	1.5829	4.1959	4.8715	4.9717	319.4***	63.22**	26.81	38.90

This table presents results from three cointegration tests, namely Kao (1999), Maddala and Wu (1999), and Pedroni (1999, 2004). The Pedroni and Kao are single-equation tests while the Maddala and Wu cointegration test uses a system of equations. They all test the null of no cointegration. To examine the stationarity of the residuals, Kao uses the standard ADF test while Pedroni uses a variety of tests, including the ADF test. For the Maddala and Wu test, we report the trace test to indicate the number of cointegration relations among variables of a system. Relationships in five models (1–5) (in column 1) were tested region-wise, after incorporating portfolios developed under the regionally biased strategy. The long-term relationship depicted in Eq. (1): ${P}_{it}={\delta }_{1i}+ {\theta }_{1i}{P}_{jt}+{\mu }_{it}$;in Eq. (2): ${P}_{it}={\delta }_{1i}+ {\theta }_{1i}{P}_{jt}+ {\theta }_{2i}{S\&P500}_{it}+{\mu }_{it}$ in Eq. (3) as ${P}_{it}={\delta }_{1i}+ {\theta }_{1i}{\Delta P}_{j,r-i,t }+ {\theta }_{2i}{S\&P500}_{it}+{\theta }_{3i}{Brent}_{it}+{\mu }_{it}$; in Eq. (4): ${P}_{it}={\delta }_{1i}+ {\theta }_{1i}{\Delta P}_{j,r-i,t }+ {\theta }_{2i}{S\&P500}_{it}{+ \theta }_{3i}{sentiments}_{it}+{\mu }_{it};$ and finally in Eq. (5): ${P}_{it}={\delta }_{1i}+ {\theta }_{1i}{\Delta P}_{j,r-i,t }+ {\theta }_{2i}GFC*{S\&P500}_{it}+{\theta }_{3i}NGFC*{S\&P500}_{it}{+\mu }_{it}$. Here, ${P}_{it}$ is each of the six Latin American nations; ${\Delta P}_{j,r-i,t}$ is the yellow portfolio of MSCI of other corresponding correlated emerging and frontier countries of the Asian, Latin American, MENA and CEE markets; the yellow colour portfolio highlight portfolio of each region with *i*) stock having correlation less than zero; ${S\&P500}_{it}$ is the price index to the US market, ${Brent}_{it}$ is international Brent oil price series and ${sentiments}_{it}$ is the global sentiment index represented by Baker and Wurgler (2006) sentiment index. *, **, *** denotes significance at 10, 5, and 1 percent or better respectively

**Table 2:** Panel cointegration test results relating to correlation based international portfolios: a summary

	Asia			LA			MENA			CEE		
	1	2	3	1	2	3	1	2	3	1	2	3
*Model 1*												
Yellow portfolio	*						*					
Blue portfolio	*			*			*		*	*		
Purple portfolio	*		*	*		*	*		*	*		*
Green portfolio						*	*		*	**–**		
Red portfolio	*					*	*		*	*		
*Model 2*												
Yellow portfolio	*											
Blue portfolio	*						*					
Purple portfolio	*						*			*	*	
Green portfolio	*						*		*			
Red portfolio	*						*					
*Model 3*												
Yellow portfolio	*	*		*	*		*	*			*	
Blue portfolio	*	*		*	*		*	*		*	*	
Purple portfolio	*	*		*	*		*	*		*	*	*
Green portfolio	*	*		*	*		*	*		*	*	
Red portfolio	*	*	*	*	*	*	*	*	*	*	*	
*Model 4*												
Yellow portfolio	*						*					
Blue portfolio	*						*					
Purple portfolio	*						*			*	*	*
Green portfolio	*						*		*			
Red portfolio	*		*			*	*		*			
*Model 5*												
Yellow portfolio	*						*					
Blue portfolio	*			*								
Purple portfolio	*			*			*			*		*
Green portfolio	*			*			*					
Red portfolio	*		*	*		*	*					

For the four emerging market groups, this table summarizes results (significant at the 5% or better) from three cointegration tests: (1) Kao (1999); (2) Maddala and Wu (1999); and (3) Pedroni (1999, 2004). The summaries relate to relationships depicted in models (1–5). Each portfolio in column 1 comprises of (*j*) markets based on the correlation between (*i*) nations from one of the four regions and (*j*) markets from all four regions (excluding *i*). Blue portfolio covers correlation between (*i*) and (j) markets of 0.2 or lower; Purple portfolio captures correlation between 0.2 and 0.3; Green portfolio covers correlation between 0.4 and 0.5; the Red portfolio stock consists of correlations of 0.6 or more

More importantly, the three cointegration tests show that, on average, across the four emerging market groups, namely Asia, CEE, MENA, and Latin America, the unconditional correlation between the MSCI data of domestic and foreign emerging markets does not appear to matter for the long-term cointegration relationship between the above data series (Table 1, Additional file 1: Table S4-1 to S4-4). These cointegration results imply that investing in markets with low correlation (which indicates high diversification gains in the short term) with the domestic market does not imply no comovement in the long term (or high long-term gains). In other words, diversification gains based on unconditional correlations do not pave the way for similar long-term gains. This result is consistent with the literature, which examines pairwise relationships between equity markets and shows that short-term correlations between two markets do not always coexist with long-term cointegration of the same markets (see detailed review in Narayan and Rehman 2021).

This analysis teaches us that selecting the yellow or blue portfolio (with low-correlated MSCIs) to improve short-term diversification gains does not always translate into long-term diversification gains. Coincidently, our results suggest that understanding long-term comovement is critical for investors who prefer to hold their portfolio investments for the long term.

Second, it appears that oil price movements, a source of global shocks, may be critical in bringing the emerging markets of Asia, Latin America, MENA, and CEE together in the long run. Model (3) with oil price provides a stronger case for cointegration than other models (1–2 and 4–5) without oil price in all four regions. This finding is consistent across the four regional groups (see Additional file 1: Tables S4-1 to S4-4). We discovered some Asian studies that find this finding. According to Zhu et al. (2014), the short-term link between oil prices and the Asian stock market was stronger after the GFC than before. According to Batten et al. (2017), hedging energy price risks will result in time-varying (short-term) gains for Asian portfolio investors. Our findings suggest that investors in all four regions can benefit from long-term diversification gains by hedging against changes in oil prices.

### VECM results

We estimate VECM versions of models (1)–(5) for each region where cointegrating relationships were found to be robustly present. All variables in the VECMs correspond to models (1)–(5), but they appear in the first difference form and include a one-lagged ECT (*ECT*) extracted from the corresponding long-term models (1)–(5). The one-period lagged ECT must be negative and significant for the VECM framework to show a stable equilibrium relationship. We then add a 0–3 lag structure to all variables (except the dummy variables) and use the standard information criteria to select the best model. Additional file 1: Tables S5-1 to S5-4 show the VECM results for each group in greater detail. Model (3) is the most robust model from the cointegration analysis because it is identified as having a cointegration relationship by all three tests. Hence in Table 4, we show model (3) for the yellow portfolio, whereas in Table 5, we show model (3) for all other portfolios. The results shed light on the relationship between each of the four emerging market groups and the rest of the emerging markets (*j*), as organized by their correlations with an (*i*) emerging market in the group. Although the effects of the portfolios are heterogeneous for each group, we find some clear patterns in terms of other independent variables’ influence on emerging market returns. These are as follows. The one-period lagged ECT are found to be insignificant in the cases of Asia, MENA, and CEE when the yellow portfolio is examined as an independent variable (Table 3, Additional file 1: Table S5-1 to S5-4). For all four portfolios, the one-lagged ECT is significant but positive for Asian markets (Table 4, Additional file 1: Table S5-2). This is also found in all CEE panels, except when the yellow portfolio is used (Table 4, Additional file 1: Table S5-4). This finding suggests that the long-term relationship between Asia’s emerging markets and the VECM regressors is rather unstable. Furthermore, depending on the international portfolio, this is also true for CEE and, on occasion, MENA nations.

**Table 3:** VECM Test: Yellow international portfolios by region

	Intercept	Δ Portfolio ret (-1)	Δ Portfolio ret (-2)	Δ Portfolio ret (-3)	Δ SP500 (-1)	Δ SP500 (-2)	Δ SP500 (-3)	Δ Brent Oil (-1)	Δ Brent Oil (-2)	Δ Brent Oil (-3)	Dummy GFC	Dummy NGFC	ECT (-1)
Latin America	7.4263*** (1.9230)	0.0588 (0.0634)	0.0139 (0.0644)	0.0637 (0.0620)	− 0.0336 (0.1323)	− 0.0840 (0.1386)	− 0.1957 (0.1342)	− 0.3906 (0.2957)	− 0.6019** (0.3163)	0.3076 (0.2683)	–	–	− 0.0055 (0.0048)
Asia	1.9367*** (0.3361)	− 0.0002 (0.0019)	0.0021 (0.0019)	− 0.0010 (0.0019)	0.7585*** (0.0221)	0.0831*** (0.0223)	0.0234 (0.0220)	− 0.0547 (0.0509)	− 0.0071 (0.0539)	–	–	–	–
MENA	− 0.8131*** (0.1170)	− 0.0012*** (0.0005)	0.0010** (0.0005)	− 0.0001 (0.0005)	0.5397*** (0.0079)	0.0158** (0.0081)	0.0178** (0.0078)	− 0.0465*** (0.0164)	− 0.0066 (0.0165)	–	–	–	–
CEE	2.2903* (1.3375)	− 0.0108** (0.0147)	0.0003 (0.9572)	0.0008 (0.0047)	2.3326*** (0.0855)	0.2791*** (0.0867)	0.0988 (0.0850)	0.1265 (0.2093)	0.7026*** (0.2303)	0.3924** (0.2075)	–	–	–

This table presents a VECM model for four emerging market groups. We only present model 3: ${\Delta P}_{i,r,t}={\delta }_{2i}+ {\theta }_{1i}{\sum }_{k=1}^{n}\Delta {P}_{j,r-i,t-k }+ {\theta }_{2i}{{\sum }_{k=1}^{n}{\Delta BRENT}_{it-k}+}{\theta }_{3i}{\sum }_{k=1}^{n}\Delta {S\&P500}_{it-k} {+{\delta }_{1i}{ECT}_{it-1}+\epsilon }_{it.}$ For the other models see Additional file 1: Table S5-1 to S5-4. Here, ${\Delta P}_{i,r}$ is a vector of returns of emerging markets, *i,* in region *r*; ${\Delta P}_{j,r-i,t}$ (columns 4–6) is a vector of returns of *j* markets in the *yellow* portfolios, excluding *i*. ${Brent}_{it}$ is Brent oil price series; and ${S\&P500}_{it}$ is the price index of the US market. These variables appear in first differenced form, represented by $\Delta$. $\delta$ and $\theta s$ are the parameters to be estimated. The error correction term (*ECT*), which is one lag of the residual from Eq. (1) if significant and negative, confirms a stable long-term relationship between the variables identified. The lag structure for the model is chosen by minimizing the Schwarz Information Criteria. Values in parenthesis are standard errors. *, **. And *** denotes level of significance at 10%, 5% and 1%, respectively

**Table 4:** VECM test results: correlation based international portfolios

	Intercept	${\Delta P}_{j,S1r-i,t-1}$	${\Delta P}_{j,S1r-i,t-2}$	${\Delta P}_{j,S1r-i,t-3}$	$\Delta {S\&P500}_{it-1}$	$\Delta {S\&P500}_{it-2}$	$\Delta {S\&P500}_{it-3}$	${\Delta Brent}_{it-1}$	${\Delta Brent}_{it-2}$	${\Delta Brent}_{it-3}$	${ECT}_{it-1}$
*Panel 1. LA*											
Blue	0.0005* (0.0001)	− 0.0079 (0.0078)	− 0.0111 (0.0082)	0.0090 (0.0074)	0.0385* (0.0110)	− 0.0238* (0.0113)	0.0305* (0.0110)	0.0110* (0.0054)	− 0.0020 (0.0054)	0.0068 (0.0054)	0.0001* (0.0000)
Purple	0.0007* (0.0002)	− 0.0074 (0.0163)	− 0.0091 (0.0162)	0.0392* (0.0158)	0.0364* (0.0175)	− 0.0272 (0.0176)	0.0270 (0.0172)	0.0102 (0.0087)	− 0.0017 (0.0086)	0.0061 (0.0087)	− 0.0001* (0.0000)
Green	0.0007* (0.0001)	− 0.0031 (0.0053)	0.0013 (0.0050)	0.0084* (0.0049)	0.0537* (0.0097)	− 0.0461* (0.0097)	0.0391* (0.0095)	0.0070 (0.0048)	0.0047 (0.0048)	0.0600* (0.0048)	− 0.0001* (0.0000)
Red	0.0007* (0.0001)	0.0005 (0.0029)	− 0.0021 (0.0029)	0.0048* (0.0027)	0.0185* (0.0035)	0.0244* (0.0035)	0.0125* (0.0035)	− 0.0001 (0.0001)	0.0001 (0.0001)	0.0002* (0.0001)	− 0.0001* (0.0000)
*Panel 2. Asia*											
Blue	0.3417 (0.1751)	− 0.0039* (0.0009)	0.0001 (0.0008)	− 0.0008 (0.0009)	0.6708* (0.0118)	0.0488* (0.0117)	0.0366* (0.0116)	− 0.0421* (0.0246)	− 0.0160 (0.0247)	–	0.8510* (0.1929)
Purple	3.6916* (0.2968)	− 0.0101* (0.0021)	− 0.0001 (0.0021)	0.0004 (0.0021)	0.7301* (0.0200)	0.2000* (0.0199)	0.0494* (0.0199)	− 0.0863* (0.0401)	− 0.0183 (0.0398)	–	3.5056* (0.2613)
Green	2.8158* (0.1748)	− 0.0007 (0.0007)	− 0.0005 (0.0007)	− 0.0015* (0.0008)	0.4361* (0.0118)	0.2276* (0.0117)	0.0255* (0.0117)	− 0.0338 (0.0237)	− 0.0174 (0.0236)	–	2.8418* (0.1517)
Red	1.7905* (0.1302)	0.0075* (0.0005)	0.0021* (0.0005)	0.0007 (0.0005)	0.8525* (0.0088)	0.5455* (0.0088)	0.1385* (0.0087)	− 0.0573* (0.0180)	0.0819* (0.0180)	–	1.5641* (0.1033)
*Panel 3. MENA*											
Blue	− 0.0001 (0.0000)	− 0.0007 (0.0025)	0.0011 (0.0024)	0.0038 (0.0024)	0.0002* (0.0000)	0.0001* (0.0000)	0.0001* (0.0000)	0.0001 (0.0000)	0.0001 (0.0000)	–	− 0.0011* (0.0003)
Purple	0.3195 (0.1387)	0.0022 (0.0009)	0.0019 (0.0009)	0.0014 (0.0009)	0.2587* (0.0093)	0.1157* (0.0093)	0.0723* (0.0092)	0.1140* (0.0195)	0.0426* (0.0197)	–	0.0017 (0.0047)
Green	0.4716* (0.1176)	0.0011* (0.0005)	0.0005 (0.0004)	0.0013* (0.0005)	0.3280* (0.0080)	0.1050* (0.0080)	0.0626* (0.0079)	0.0696* (0.0163)	0.0576* (0.0164)	–	0.0521 (0.1030)
Red	0.4733* (0.0493)	0.0014* (0.0002)	0.0011* (0.0002)	0.0004* (0.0002)	0.2290* (0.0334)	0.0831* (0.0033)	0.0633* (0.0033)	0.0463* (0.0069)	0.0344* (0.0069)	–	0.1202* (0.0412)
*Panel 4. CEE*											
Blue	0.3417 (0.1751)	− 0.0039* (0.0009)	0.0001 (0.0008)	− 0.0008 (0.0009)	0.6708* (0.0118)	0.0488* (0.0117)	0.0366* (0.0116)	− 0.0421* (0.0246)	− 0.0160 (0.0247)	–	0.8510* (0.1929)
Purple	3.6916* (0.2968)	− 0.0101* (0.0021)	− 0.0001 (0.0021)	0.0004 (0.0021)	0.7301* (0.0200)	0.2000* (0.0199)	0.0494* (0.0199)	− 0.0863* (0.0401)	− 0.0183 (0.0398)	–	3.5056* (0.2613)
Green	2.8158* (0.1748)	− 0.0007 (0.0007)	− 0.0005 (0.0007)	− 0.0015* (0.0008)	0.4361* (0.0118)	0.2276* (0.0117)	0.0255* (0.0117)	− 0.0338 (0.0237)	− 0.0174 (0.0236)	–	2.8418* (0.1517)
Red	1.7905* (0.1302)	0.0075* (0.0005)	0.0021* (0.0005)	0.0007 (0.0005)	0.8525* (0.0088)	0.5455* (0.0088)	0.1385* (0.0087)	− 0.0573* (0.0180)	0.0819* (0.0180)	–	1.5641* (0.1033)

This table presents a VECM model for four emerging market groups. We only present model 3: ${\Delta P}_{i,r,t}={\delta }_{2i}+ {\theta }_{1i}{\sum }_{k=1}^{n}\Delta {P}_{j,r-i,t-k }+ {\theta }_{2i}{{\sum }_{k=1}^{n}{\Delta BRENT}_{it-k}+}{\theta }_{3i}{\sum }_{k=1}^{n}\Delta {S\&P500}_{it-k} {+{\delta }_{1i}{ECT}_{it-1}+\epsilon }_{it.}$ For the other models see Additional file 1: Tables S5-1 to S5-4. Here, ${\Delta P}_{i,r}$ is a vector of returns of emerging markets, *i,* in region *r*; ${\Delta P}_{j,r-i,t}$ (columns 4–6) is a vector of returns of *j* markets in the *Blue-Red* portfolios, excluding *i*. Blue portfolio captures correlation between (*i*) and (*j*) markets of 0.2 or lower; Purple portfolio captures correlation between 0.2 and 0.3; Green portfolio covers correlation between 0.4 and 0.5; the Red portfolio stock consists of correlations of 0.6 or more. ${Brent}_{it}$ is Brent oil price series; and ${S\&P500}_{it}$ is the price index of the US market. These variables appear in first differenced form, represented by $\Delta$. $\delta$ and $\theta s$ are the parameters to be estimated. The error correction term (*ECT*), which is one lag of the residual from Eq. (1) if significant and negative, confirms a stable long-term relationship between the variables identified. The lag structure for the model is chosen by minimizing the Schwarz Information Criteria. Values in parenthesis are standard errors. * denotes level of significance at 5% or better

Our findings on Asia are consistent with cointegration studies on Asia. Previous research on cointegration has yielded varying results at different times. For example, studies covering the Asian financial crisis period (1997–1998) or the GFC (2007–2008) generally show cointegration, whereas studies outside of the two events generally show no cointegration. Furthermore, some studies show varying degrees of (short-term) correlation (or coupling and decoupling, see Dooley and Hutchison 2009) of Asian markets with: the U.S. during the GFC and NGFC period (Dooley and Hutchison 2009; Narayan et al. 2014); and oil price during the GFC and NGFC period (Zhu et al. 2014). Because our study spans the years 2000–2016, our VECM models detected short-term engaging and disengaging behavior that our chosen cointegration tests did not. Considering these new VECM results, we are finding a case of an unstable long-term relationship between Asia, the high-to-low correlation portfolios, and other regressors. Therefore, we take this volatile long-term behavior as our main finding for Asia and argue that Asian investors can diversify their portfolios within the emerging market region. The correlation-based portfolios have no effect on the VECM results for Asia, which is consistent with the cointegration tests.

In the long term, the CEE markets have an unstable relationship with all but the negative-to-least correlated (yellow) portfolio. The region’s unstable long-term relationship is also not surprising, given the prevalence of shocks that had varying effects on short-term integration during our study period. According to studies, the E.U. accession and the GFC facilitated integration among CEE nations, implying that CEE integration with non-CEE nations deteriorated due to these events. For example, Demian (2011) and Caporale and Spagnolo (2012) show increased integration between CEE stock markets following E.U. accession. Bieńkowski et al. (2014) discovered a positive relationship between the GFC and the correlation of three CEE stock markets (Poland, the Czech Republic, and Hungary). However, the Eurozone sovereign debt crisis shifted investor attention away from CEE, resulting in less integration among CEE nations while increased integration with crisis-unaffected nations (Narayan et al. 2020). However, some authors find no significant link between the CEE markets and other international markets (Linne 1998; Scheicher 2001).

The one-period lagged *ECT* is primarily negative and significant for the two other regions: Latin America (in the case of all portfolios) and MENA (depending on the models). This means that the relationships depicted in the VECM for the two regions are stable. In other words, following a model shock, the returns of country *i* revert to the long-term equilibrium level of stock prices. As a result, we will now focus on three regions: CEE (yellow portfolio only), Latin America (all portfolios), and MENA (blue and/or purple portfolios). Individual MSCIs in these three regions also respond significantly to short-term changes in international emerging market returns. The effect of international markets is felt a day after the shock in CEE and MENA. Only in Latin America do international emerging markets have a delayed effect.

The S&P 500 has consistently been found to have a positive and significant effect on emerging markets (*i*). The S&P 500 is also found to be a better predictor of emerging market (*i*) returns than other emerging markets (*j*). We also find that the impact of the S&P 500 on emerging market groups is greater during the U.S. GFC than during the NGFC period.

Oil prices are found to harm CEE stock market returns, but a positive effect in Latin America and MENA groups. Because Latin America and MENA nations are primarily net exporters of crude oil and/or refined petroleum, this result is not surprising. The effects of oil prices are weaker in all four regions than they are in the S&P 500.

### Long-term results

Given the strong case for cointegration between emerging markets and other control variables, we examine the long-term relationships depicted in models (1)–(5). To determine the long-term relationships between cointegrated variables, the dynamic OLS estimation method is used. Models (1)–(5) regress domestic emerging market MSCI (*i*) against the *j* portfolios and other regressors (S&P 500, oil price, investor sentiment, S&P 500 during GFC (GFC), and/or S&P 500 during NGFC). The results are presented in Additional file 1: Tables S6-1 to S6-4. Table 5 shows each region’s long-term results for the five models for the yellow portfolio. Table 6 displays model (3) by four regions. The key results are as follows.

**Table 5:** Long-term regression results: yellow international portfolios

	${P}_{jt}$	SP500	Brent oil	Sentiment	Dummy GFC	Dummy NGFC
*Latin America*						
Model 1	0.0003 (0.0548)	–	–	–	–	–
Model 2	− 0.0213 (0.0570)	0.1719 (0.1243)	–	–	–	–
Model 3	− 0.0461 (0.0574)	0.1289 (0.1246)	− 0.2257 (0.2542)	–	–	–
Model 4	− 0.0211 (0.0570)	0.1693 (0.1243)	–	4.2674 (3.1271)	–	–
Model 5	− 0.0213 (0.0570)	–	–	–	0.1414 (0.1582)	0.1784 (0.1261)
*Asia*						
Model 1	0.0067* (0.0019)	–	–	–	–	–
Model 2	0.0052* (0.0018)	0.7693* (0.0217)	–	–	–	–
Model 3	0.0049* (0.0019)	0.7662* (0.0219)	0.2300* (0.0454)	–	–	–
Model 4	0.0052* (0.3288)	0.7694* (0.0217)	− 0.1862 (0.5640)	–	–	–
Model 5	0.0052 (0.0018)	–	–	–	0.7650* (0.0254)	0.7704* (0.0220)
*MENA*						
Model 1	0.0162* (0.0005)	–	–	–	–	–
Model 2	0.0139* (0.0005)	0.4010* (0.0076)	–	–	–	–
Model 3	0.0135* (0.0005)	0.3968* (0.0077)	0.0868* (0.0150)	–	–	–
Model 4	0.0139* (0.0005)	0.4010* (0.0076)	–	− 0.2373 (0.1961)	–	–
Model 5	0.0139* (0.0005)	–	–	–	0.4359* (0.0087)	0.3935* (0.0077)
*CEE*						
Model 1	0.0191* (0.0042)	–	–	–	–	–
Model 2	0.0052 (0.0042)	2.9582* (0.0755)	–	–	–	–
Model 3	0.0048 (0.0043)	2.9582* (0.0768)	0.2373 (0.1724)	–	–	–
Model 4	0.0052* (1.1755)	2.9589* (0.0756)	–	− 1.4907 (1.9816)	–	–
Model 5	0.0052 (0.0042)	–	–	–	2.9405* (0.0843)	2.9631* (0.0762)

This table presents a long-term relationship for the four emerging market groups, relating model (1): ${P}_{it}={\delta }_{1i}+ {\theta }_{1i}{P}_{jt}+{\mu }_{it}$;in model (2): ${P}_{it}={\delta }_{1i}+ {\theta }_{1i}{P}_{jt}+ {\theta }_{2i}{S\&P500}_{it}+{\mu }_{it}$ in model (3) as ${P}_{it}={\delta }_{1i}+ {\theta }_{1i}{\Delta P}_{j,r-i,t }+ {\theta }_{2i}{S\&P500}_{it}+{\theta }_{3i}{Brent}_{it}+{\mu }_{it}$; in model (4): ${P}_{it}={\delta }_{1i}+ {\theta }_{1i}{\Delta P}_{j,r-i,t }+ {\theta }_{2i}{S\&P500}_{it}{+ \theta }_{3i}{sentiments}_{it}+{\mu }_{it};$ and finally in model (5): ${P}_{it}={\delta }_{1i}+ {\theta }_{1i}{\Delta P}_{j,r-i,t }+ {\theta }_{2i}GFC*{S\&P500}_{it}+{\theta }_{3i}NGFC*{S\&P500}_{it}{+\mu }_{it}$. Here, ${P}_{it}$ is each of the six Latin American nations; ${\Delta P}_{j,r-i,t}$ is the yellow portfolio of MSCI of other corresponding correlated emerging and frontier countries of the Asian, Latin American, MENA and CEE markets with correlations between markets *i* and *js* of less than zero; ${S\&P500}_{it}$ is the price index to the US market, ${Brent}_{it}$ is international Brent oil price series and ${sentiments}_{it}$ is the global sentiment index represented by Baker and Wurgler (2006) sentiment index. * denotes level of significance at 5% or better

**Table 6:** Long-term regression results: blue-red international portfolios

	${P}_{j,S1r-i}$	SP500	Brent oil		${P}_{j,S1r-i}$	SP500	Brent oil
*Panel 1: LA*				*Panel 3: MENA*			
Blue portfolio	− 0.0128* (0.0117)	0.0011* (0.0001)	0.0126* (0.0002)	Blue portfolio	− 0.0252* (0.0080)	0.0031 (0.0030)	0.0275* (0.0011)
Purple portfolio	0.6562 (0.0209)	0.0009* (0.0001)	0.0058* (0.0004)	Purple portfolio	0.3228* (0.0045)	0.0548* (0.0019)	0.1503* (0.0035)
Green portfolio	0.5611* (0.0078)	0.0007* (0.0001)	0.0077* (0.0002)	Green portfolio	0.5234* (0.0045*)	− 0.2264* (0.0118)	0.1091* (0.0037)
Red portfolio	0.7874* (0.0043)	0.0001* (0.0000)	0.0034* (0.0001)	Red portfolio	0.6546* (0.0026)	0.2437* (0.0062)	0.1148* (0.0021)
*Panel 2: ASIA*				*Panel 4: CEE*			
Blue portfolio	− 0.2277* (0.0053)	1.2074* (0.0125)	0.1126* (0.0033)	Blue portfolio	− 0.0729* (0.0061)	0.8954* (0.0140)	0.2636* (0.0050)
Purple portfolio	0.4143* (0.0088)	1.2269* (0.0163)	0.0828* (0.0060)	Purple portfolio	0.4724* (0.0087)	0.1929* (0.0173)	0.1229* (0.0061)
Green portfolio	0.4383* (0.0057)	1.0317* (0.0120)	0.0857* (0.0041)	-Green portfolio	0.4560* (0.0060)	0.6143* (0.0157)	0.1883* (0.0053)
Red portfolio	0.6727* (0.0026)	0.8104* (0.0066)	0.0671* (0.0021)	Red portfolio	0.6543* (0.0021)	0.4390* (0.0051)	0.1090* (0.0017)

This table presents a long-term relationship for the four emerging market groups, relating to model (3): ${P}_{it}={\delta }_{1i}+ {\theta }_{1i}{P}_{j,S1r-i,t}+ {\theta }_{2i}{S\&P500}_{it}+{\theta }_{4i}{Brent}_{it}+{\mu }_{it}$. The dependent variable, ${\Delta P}_{i,r}$, is a vector of returns of emerging markets, *i*, in region *r*. ${\Delta P}_{j,1r-i}$ is a vector of returns of *j* markets in Blue-Red portfolios, excluding *i*. Blue portfolio captures correlation between (*i*) and (*j*) markets of 0.2 or lower; Purple portfolio captures correlation between 0.2 and 0.3; Green portfolio covers correlation between 0.4 and 0.5; the Red portfolio stock consists of correlations of 0.6 or more. ${Brent}_{it}$ is Brent oil price series; and ${S\&P500}_{it}$ is the price index of the US market. For results relating to models (1–2 and 4–5) refer to Additional file 1: Tables S6-1 to S6-4. Values in parenthesis are standard errors. * denotes level of significance at 5% or better

First, we find that the long-term relationships depend strongly on the portfolios examined. Highly correlated (*j*) emerging markets have stronger long-term effects on (*i*) emerging markets than the less correlated (*j*) emerging markets (see model (1), Additional file 1: Tables S6-1 to S6-4). According to conventional wisdom, a portfolio of highly correlated (*i* and *j*) emerging markets is more vulnerable to shocks from (*j*) markets than a portfolio of lowly correlated (*i* and *j*) emerging markets. This finding is applicable to all regions examined and consistent across all models, confirming the robustness of the models and methods used.

Second, the effects of the S&P 500 are magnified in an investment portfolio that includes less correlated (*i* and *j*) emerging markets. Similarly, the effects of the S&P500 are reduced in portfolios where foreign emerging markets (*j*) are highly correlated with domestic emerging markets (*i*). What appears to be a logically consistent finding is true for Asia, Latin America, and CEE regions, but not for MENA (see model 3, Table 6).^3^ Thus, investing in low (high) correlated (*j*) emerging markets implies greater (lesser) vulnerability to S&P 500 shocks. In other words, a weakly integrated portfolio is less able to deal with U.S. shocks. This finding implies that in times of intense negative shocks from the S&P 500, (*j*) emerging market investors will benefit from diversifying their investment portfolio with high and low correlated (*j*) emerging market MSCI, as this will reduce the S&P 500’s vulnerability. During the recent European sovereign debt crisis, Narayan et al. (2020) found increased stock market correlations between CEE nations and other emerging markets.

Third, the S&P 500’s effects are stronger during the GFC than during the NGFC. This is true and robust across all four panels (see Additional file 1: Table S6-1 to S6-4). Furthermore, as mentioned in point 2 above, for the Latin American and CEE regions, the variation in the magnitude of the effects decreases as the correlation between* i* and *j* increases, but not for the MENA region.

Fourth, the effects of oil prices are bigger (smaller) when conditioned to the least (most) correlated emerging market equities (see model 3, Table 6).

Fifth, unlike oil prices and the S&P 500, the effects of investor sentiment are stronger (weaker) when conditioned with high (low) correlated emerging market equities (see model 4, Additional file 1: Tables S6-1 to S6-4).

In terms of investment strategy, it appears that the findings revealed in points 3, 4, and 5 above suggest that a mix of very lowly and very highly correlated emerging market equities will benefit investors in combating the negative effects of investor sentiment, S&P 500, and oil price shocks. However, switching to very highly correlated MSCIs will reduce vulnerability if there is a persistently large oil shock or S&P 500 shock. If there is a persistently large investor sentiment shock, holding large amounts of the least correlated emerging market MSCIs will mitigate its effects.

The five differences in the effects of shocks between portfolios with low or high correlations are consistent across the four regions, though there are some important differences in the magnitude of the effects of the factors from one region to the next.

According to the VECM models, the S&P 500 has always had a greater short-term impact than emerging markets or oil prices. This is found to be true in long-term models for Latin America (Table 6, panel 1) and CEE (Table 6, panel 4). However, we find differences in the size effects of the S&P 500 compared to oil prices and emerging markets in the other regions.:

1. In the case of MENA, the (*j*) emerging markets effects dominate. The (*j*) emerging markets and oil price effects outperform the S&P 500. For MENA.
2. For CEE, the effects of oil prices are less than those of the S&P 500 and (*j*) emerging markets. Furthermore, for the CEE group, the effects of (*j*) emerging markets are greatest when the models are conditioned to (green) portfolios with correlations of 40–50%.

In terms of regressor sign effects, we find that the long-term effects of the independent variables (the (*j*) emerging markets portfolios, S&P 500, oil price, investor sentiment, S&P 500 GFC, and S&P 500 NGFC) on the dependent variable are almost always positive. When the Latin American group is in a portfolio with its most correlated counterparts, the S&P 500 suffers a negative effect. However, the effect becomes positive when the oil price is included. The S&P 500 negatively affects the MENA group when the group is linked with its mid-range correlated emerging market partners. When oil prices or investor sentiment are included, the negative effect disappears. The least correlated emerging markets negatively impact the CEE group in all models other than model (1).

## Further analysis

Finally, we update our dataset to create portfolios for 2017–2022 based on the average conditional correlations calculated up to 2016 (Additional file 1: Table S1). To put it another way, we use the correlation strategy described in the previous section. This is analogous to building portfolios with even less knowledge. Our analysis considers the fact that investors use prior knowledge (in this case, on correlation strategy) to develop their portfolio over the period 2017–2022. We adhere to the previous methodology to estimate the correlation strategy’s long-term benefits. Initially, we find that nearly all portfolios (${P}_{i}$ and ${P}_{j}$) across regions, and other variables, such as oil price and sentiment, are stationary over the period 2017–2022.^4^ This indicates that most portfolios developed using the past correlation matrix are unsuitable for testing the long-term benefits of the correlation strategies used to develop the various portfolios, because these portfolios were affected by transitory shocks. The methodology can only test Asia’s Yellow and MENA Blue portfolios. Interestingly, these are the two portfolios with the least correlated assets. The previous models (1) and (2) can be used for these two portfolios as well as one other variable, the S&P 500. For the Asian and MENA portfolios, we find that both models (1) and (2) are cointegrated.^5^ This suggests that the benefits of the correlation strategy disappear in the long-term, which is consistent with the main findings of the previous analysis.

## Conclusion

To date, many studies have addressed whether investors will benefit from international portfolio diversification in emerging market nations without considering the investment strategies used. Markowitz (1952) recommended using low-correlated assets to control portfolio risk, which is commonly used to rebalance portfolios. However, its advantages for long-term investors remain poorly known.

This study uniquely examines the long-term diversification gains of international portfolios in four regions by considering a commonly used rebalancing/hedging strategy. It evaluates emerging markets using MSCI-based investment portfolios and a high-to-low correlation strategy, in which equity portfolios for each country are developed based on the correlation between the domestic stock market and four emerging markets (Asia, Latin America, MENA, and CEE). Our correlation-based portfolios are identified by color, with the blue portfolio having the lowest correlations and the red portfolio having the highest correlations between emerging equity markets. We examine the long-term diversification gains under various scenarios and conditions related to economic and financial shocks affecting emerging markets. Our key findings and their implications for long-term investors are summarized below.

While there is a long-term relationship between the (*i*) emerging markets and (*js*) correlation-based portfolios, the long-term coefficients show that the blue and purple portfolios (which contain relatively low-correlated assets) have a lower impact on domestic emerging markets than our two portfolios with highly correlated emerging markets (the green and red portfolios). This finding indicates that portfolios with lowly correlated international emerging markets (blue and purple) outperform portfolios with highly correlated international emerging markets (red and green).

Asia and CEE are found to be distinct from the other two regions, as all models fail to work under the VECM framework under both or one of the investment strategies. This could suggest that the international portfolios developed here provide more diversification opportunities for Asian and CEE investors in the long run than investors from other regions. Overall, the cointegration test results are unaffected by whether the portfolios are less or more correlated. However, the long-term regression analysis of cointegrated variables suggests that low-correlated portfolios outperform high-correlated portfolios in terms of long-term diversification gains.

Second, it appears that oil prices are driving the long-term comovement of emerging markets. This study looks at five different models, starting with model (1), which only explores the effects of (*i*) emerging markets on (*j*) emerging markets. Each subsequent model (models (2)–(5)) added one new factor. This setup proved useful for determining the robustness of model (1) under various economic and financial conditions and determining the importance of additional variable(s) in influencing the outcomes. Through this latter process, we find that model (3), which was supplemented with the Brent oil price, played an important role in establishing a stable long-term comovement between emerging markets. As a result, it appears that the price of oil is an important factor driving long-term comovement.

Third, we find that the sources of international shocks that have the greatest long-term impact on emerging markets differ depending on the investors’ portfolios and regions of residence. Asian or CEE investors with the least correlated *j* market-based portfolios are the most vulnerable to S&P 500 performance. Alternatively, investors in Latin America or MENA with the least correlated portfolio of emerging markets are the most vulnerable to oil price fluctuations. Investors in CEE, MENA, and Latin America with more correlated emerging market portfolios (purple and red portfolios) are particularly vulnerable to highly correlated foreign emerging markets.

Overall, this study presents an empirical method for assessing the long-term benefits of a popular portfolio investment strategy. We applied the methodology to emerging and frontier stock markets and documented long-term portfolio investment behavior during one of the most severe financial crises, the GFC. We also provide additional analysis that introduced more flaws in knowledge when building investment portfolios. Limited portfolios examined using the long-term analysis behaved similarly to those depicted in the previous analysis.

This study can be expanded in several ways. First, we can apply our methods to the COVID-19 pandemic. Second, the models may include additional factors such as developed markets other than the S&P 500. Third, we develop our portfolios by averaging the correlation over the sample period (Additional file 1: Table S2), assuming that market correlations are time invariant. While this assumption allowed us to keep balanced panels for our empirical analyses, it would be a constraint if the variation in the correlation across time was significant. Future research may consider calculating time-varying correlations and, if necessary, incorporating time-varying correlations into portfolio development by allowing market pairs *i* and *j* to exist in different portfolios at different times. Unbalanced panels can thus be used in the analysis.

## Supplementary Information

**Additional file 1**. Supplementary materials.

## Data Availability

The datasets used during the current study are available from the corresponding author on reasonable request.

## Abbreviations

CEE: Central and Eastern Europe
MENA: Middle East and North Africa
VECM: Vector error correction model
GFC: Global financial crisis
ARCH: Autoregressive conditional heteroskedasticity model
GARCH: Generalized autoregressive conditional heteroskedasticity
MSCI: Morgan Stanley Capital International
GCC: Gulf Cooperation Council
EFA: Emerging and frontier Asian
DJIA: Dow Jones Industrial Average
LSDV: Least squares dummy variable
OLS: Ordinary least squares
VAR: Vector autoregressive
EIA: Energy information administration
ECT: Error correction terms
NGFC: Non-GFC

## Footnotes

1 Our models are adopted from Narayan and Rehman (2021). The authors used the models to test regional portfolio without paying attention to correlation strategy covered here.

2 We cover a period around the 2008 financial crisis for key emerging and frontier stock markets. The total number of observations by regions and portfolios are added as part of supplementary material in Additional file 1: Table S7.

3 Note this pattern seems to apply only with portfolios with correlations above zero. Table 2 which captures the yellow portfolio with negative correlated assets fails to bind by this logic.

4 This means that models (3) and (4) could not be applied. Further model (5) is irrelevant as between 2017 and 2022 we could not allow for the GFC.

5 These results are available on request from the authors.
