# Supplementary material for: Is a correlation-based investment strategy beneficial for long-term international portfolio investors?
Source: Financ Innov. 2023 Mar 9;9(1):64. doi: 10.1186/s40854-023-00471-9 (PMC9995254; doi:10.1186/s40854-023-00471-9)
Supplement: Supplementary file 1 — Additional file 1. Supplementary materials. [file 40854_2023_471_MOESM1_ESM.docx]

**Supplementary material**

**Table S1** Unconditional Correlations between emerging market returns by regions

| ***Panel A***: Latin America | Argentina | Brazil | | Chile | | Colombia | | | Mexico | | | Venezuela | | |  |  |  |  |  |  |  |  |
| --- | --- | --- | --- | --- | --- | --- | --- | --- | --- | --- | --- | --- | --- | --- | --- | --- | --- | --- | --- | --- | --- | --- |
| Argentina | 1 |  | |  | |  | | |  | | |  | | |  |  |  |  |  |  |  |  |
| Brazil | 0.397* | 1 | |  | |  | | |  | | |  | | |  |  |  |  |  |  |  |  |
| Chile | 0.539* | 0.915* | | 1 | |  | | |  | | |  | | |  |  |  |  |  |  |  |  |
| Colombia | 0.436* | 0.868* | | 0.946* | | 1 | | |  | | |  | | |  |  |  |  |  |  |  |  |
| Mexico | 0.714* | 0.858* | | 0.941* | | 0.899* | | | 1 | | |  | | |  |  |  |  |  |  |  |  |
| Venezuela | 0.505* | 0.437* | | 0.545* | | 0.509* | | | 0.624* | | | 1 | | |  |  |  |  |  |  |  |  |
| ***Panel B***: Asia | Pakistan | India | Bangladesh | | | | China | | | Sri Lanka | | | Indonesia | | Korea | | Malaysia. | | Philippines | | Thailand | |
| Pakistan | 1 |  |  | | | |  | | |  | | |  | |  | |  | |  | |  | |
| India | 0.876* | 1 |  | | | |  | | |  | | |  | |  | |  | |  | |  | |
| Bangladesh | 0.353* | 0.462* | 1 | | | |  | | |  | | |  | |  | |  | |  | |  | |
| China | 0.457* | 0.614* | 0.261* | | | | 1 | | |  | | |  | |  | |  | |  | |  | |
| Sri Lanka | 0.761* | 0.859* | 0.576* | | | | 0.403* | | | 1 | | |  | |  | |  | |  | |  | |
| Indonesia | 0.855* | 0.914* | 0.528* | | | | 0.490* | | | 0.884* | | | 1 | |  | |  | |  | |  | |
| Korea | 0.743* | 0.903* | 0.435* | | | | 0.604* | | | 0.836* | | | 0.877* | | 1 | |  | |  | |  | |
| Malaysia | 0.114* | 0.051* | -0.092* | | | | 0.242* | | | -0.003 | | | -0.022 | | -0.017 | | 1 | |  | |  | |
| Philippines | 0.852* | 0.853* | 0.491* | | | | 0.472* | | | 0.827* | | | 0.881* | | 0.757* | | 0.286* | | 1 | |  | |
| Thailand | 0.849* | 0.879* | 0.400* | | | | 0.386* | | | 0.862* | | | 0.917* | | 0.849* | | -0.016 | | 0.867* | | 1 | |
| ***Panel C***: MENA | Egypt | Greece | Jordan | | Kuwait | | | Lebanon | | | Oman | | | Kenya | | Morocco | | Tunisia | |  |  |  |
| Egypt | 1 |  |  | |  | | |  | | |  | | |  | |  | |  | |  |  |  |
| Greece | -0.143* | 1 |  | |  | | |  | | |  | | |  | |  | |  | |  |  |  |
| Jordan | 0.720* | 0.201* | 1 | |  | | |  | | |  | | |  | |  | |  | |  |  |  |
| Kuwait | 0.688* | 0.367* | 0.903* | | 1 | | |  | | |  | | |  | |  | |  | |  |  |  |
| Lebanon | 0.856* | -0.048* | 0.818* | | 0.704* | | | 1 | | |  | | |  | |  | |  | |  |  |  |
| Oman | 0.929* | -0.096* | 0.810* | | 0.758* | | | 0.897* | | | 1 | | |  | |  | |  | |  |  |  |
| Kenya | 0.899* | -0.027 | 0.707* | | 0.722* | | | 0.783* | | | 0.858* | | | 1 | |  | |  | |  |  |  |
| Morocco | 0.697* | 0.001 | 0.495* | | 0.451* | | | 0.771* | | | 0.744* | | | 0.600* | | 1 | |  | |  |  |  |
| Tunisia | 0.724* | -0.675* | 0.280* | | 0.143* | | | 0.653* | | | 0.650* | | | 0.605* | | 0.627* | | 1 | |  |  |  |
| ***Panel D***: CEE | Hungary | Poland | Russia | | South Africa | | | Turkey | | | Croatia | | | Estonia | | Lithuania | | Romania | | Slovenia | |  |
| Hungary | 1 |  |  | |  | | |  | | |  | | |  | |  | |  | |  | |  |
| Poland | 0.889* | 1 |  | |  | | |  | | |  | | |  | |  | |  | |  | |  |
| Russia | 0.792* | 0.815* | 1 | |  | | |  | | |  | | |  | |  | |  | |  | |  |
| South Africa | 0.701* | 0.838* | 0.501* | | 1 | | |  | | |  | | |  | |  | |  | |  | |  |
| Turkey | 0.751* | 0.871* | 0.594* | | 0.958* | | | 1 | | |  | | |  | |  | |  | |  | |  |
| Croatia | 0.703* | 0.682* | 0.809* | | 0.277* | | | 0.301* | | | 1 | | |  | |  | |  | |  | |  |
| Estonia | 0.893* | 0.925* | 0.677* | | 0.858* | | | 0.871* | | | 0.557* | | | 1 | |  | |  | |  | |  |
| Lithuania | 0.922* | 0.920* | 0.722* | | 0.788* | | | 0.802* | | | 0.656* | | | 0.967* | | 1 | |  | |  | |  |
| Romania | 0.916* | 0.922* | 0.793* | | 0.698* | | | 0.723* | | | 0.771* | | | 0.913* | | 0.955* | | 1 | |  | |  |
| Slovenia | 0.210* | 0.155* | 0.346* | | -0.254* | | | -0.274* | | | 0.770* | | | 0.019 | | 0.161* | | 0.307* | | 1 | |  |

Notes: * denotes level of significance at 5% or better.

**Table S2** International equity portfolios for individual emerging market by correlations

| Portfolios | Yellow | Blue | Purple | Green | Red |
| --- | --- | --- | --- | --- | --- |
| Correlation range | -100% - 0.1% | 0% - 20% | 21% - 39% | 40% - 59% | 60% - 100% |
| Argentina | Greece, Slovenia | Croatia, Kuwait, Jordan, Malaysia, Russia, Morocco | Lebanon, Oman | Bangladesh, Kenya, China, Brazil, Colombia, Romania, Venezuela, Egypt, Hungary, Poland, Chile | Lithuania, Korea, Tunisia, Estonia, Sri Lanka, Turkey, Thailand, Mexico, Indonesia, Philippines, India, South Africa, Pakistan |
| Brazil | Greece, Malaysia | Bangladesh | - | Argentina, China, Venezuela, Philippines, Croatia, Pakistan, Kuwait | Morocco, Thailand, Sri Lanka, Jordan, Indonesia, S. Africa, Lithuania, India, Romania, Estonia, Tunisia, Turkey, Russia, Oman, Kenya, Hungary, Egypt, Poland, Lebanon, Korea, Mexico, Colombia, Chile |
| Chile | Greece, Slovenia, Malaysia | - | Croatia, Kuwait | China, Bangladesh, Jordan, Argentina, Venezuela | Morocco, Romania, Pakistan, Russia, Philippines, Kenya, Lithuania, Oman, Hungary, Lebanon, Egypt, Estonia, Poland, Thailand, S. Africa, Sri Lanka, India, Indonesia, Turkey, Tunisia, Korea, Brazil, Mexico, Colombia |
| Colombia | Greece, Slovenia, Malaysia | Croatia | China, Bangladesh, Kuwait | Argentina, Morocco, Jordan, Venezuela | Romania, Pakistan, Philippines, Hungary, Russia, Lithuania, Kenya, Estonia, Lebanon, Egypt, Oman, Poland, India, Sri Lanka, South Africa, Thailand, Indonesia, Turkey, Korea, Tunisia, Brazil, Mexico, Chile |
| Mexico | Greece, Slovenia, Malaysia | - | Croatia, Kuwait | Bangladesh, China, Jordan, Morocco | Russia, Venezuela, Lebanon, Oman, Romania, Kenya, Hungary, Argentina, Lithuania, Egypt, Philippines, Pakistan, Poland, Sri Lanka, Estonia, Brazil, Thailand, Tunisia, Korea, Indonesia, India, South Africa, Colombia, Turkey, Chile |
| Venezuela | Greece, Slovenia | Malaysia, Kuwait, Croatia, Jordan | Jordan, Bangladesh, Morocco, Russia, China | Lebanon, Oman, Hungary, Romania, Brazil, Egypt, Kenya, Lithuania, Argentina, Colombia, Chile, Poland | Estonia, Philippines, Tunisia, Pakistan, Indonesia, Korea, Thailand, Sri Lanka, India, Turkey, Mexico, South Africa |
| Pakistan | Greece, Slovenia | Malaysia, Croatia, Kuwait | Jordan, Russia | Morocco, Bangladesh, Lebanon, China, Brazil, Oman | Colombia, Venezuela, Kenya, Chile, Hungary, Romania, Egypt, Poland, Tunisia, Korea, Lithuania, Sri Lanka, Mexico, Estonia, Turkey, Thailand, Philippines, Indonesia, India, Argentina, South Africa |
| India | Greece, Slovenia | Malaysia | Kuwait | Croatia, Jordan, Bangladesh | Russia, Venezuela, Morocco, China, Lebanon, Brazil, Romania, Oman, Kenya, Colombia, Hungary, Argentina, Lithuania, Chile, Estonia, Egypt, Poland, Philippines, Sri Lanka, Pakistan, Mexico, Thailand, Tunisia, Korea, Indonesia, Turkey, South Africa |
| Bangladesh | Greece, Slovenia, Kuwait, Croatia, Jordan, Malaysia | Croatia, Jordan, Malaysia, Romania, Kenya, Lithuania, Russia, Hungary, Brazil, Poland | China, Venezuela, Colombia | Pakistan, Argentina, Morocco, Mexico Thailand, Chile, Korea, South Africa, Turkey, India, Philippine, Indonesia | Sri Lanka, Tunisia, Bangladesh |
| China | - | Greece, Malaysia | Bangladesh, Colombia, Jordan, Venezuela | Argentina, Thailand, Sri Lanka, Chile Mexico, Brazil, Slovenia, Kuwait, Pakistan, Philippine, Indonesia, Estonia, Tunisia, Kenya, Lebanon, Turkey, Lithuania | South Africa, Russia, Romania, Oman, Hungary, Korea, India, Croatia, Egypt, Poland, Morocco |
| Sri Lanka | Greece, Slovenia | Kuwait, Croatia, Jordan | - | China, Russia, Morocco, Lebanon, Romania, Oman | Bangladesh, Hungary, Kenya, Venezuela, Brazil, Lithuania, Egypt, Argentina, Poland, Estonia, Colombia, Pakistan, Chile, Mexico, Philippines, Korea, South Africa, India, Thailand, Tunisia, Indonesia, Turkey |
| Indonesia | Greece, Slovenia | Kuwait, Croatia, Jordan | - | Russia, China, Morocco, Bangladesh, Lebanon | Venezuela, Romania, Oman, Hungary, Brazil Kenya, Lithuania, Egypt, Argentina, Colombia, Poland, Estonia, Chile, Pakistan, Mexico, Korea, Philippines, Sri Lanka, Tunisia, India, Thailand, Turkey, South Africa |
| Korea | Greece, Slovenia | - | - | Kuwait, Bangladesh, Croatia, Jordan | Venezuela, Argentina, China, Morocco, Russia, Pakistan, Lebanon, Romania, Philippines, Brazil, Kenya, Oman, Hungary, Lithuania, Colombia, Sri Lanka, Estonia, Egypt, Thailand, Chile, Mexico, Tunisia, Indonesia, Poland, South Africa, India, Turkey |

**Table S2 cont’d** International equity portfolios for individual emerging market by correlations

| Portfolios | Yellow | Blue | Purple | Green | Red |
| --- | --- | --- | --- | --- | --- |
| Correlation range | -100% - 0.1% | 0% - 20% | 21% - 39% | 40% - 59% | 60% - 100% |
| Malaysia | Columbia, Chile, Brazil, Tunisia, Mexico, Jordan, Bangladesh, Oman | Venezuela, Turkey, South Africa, India, Argentina, Egypt, Hungary, Pakistan, Estonia, Romania, Lithuania, Kenya, Poland, Croatia, Morocco, China | Philippine | Slovenia, Greece | Malaysia |
| Philippines | Greece, Slovenia | Jordan, Croatia | Russia, Malaysia | Lebanon, Brazil, Oman, China, Morocco, Romania, Bangladesh, Hungary, Kenya | Venezuela, Colombia, Lithuania, Egypt, Chile, Poland, Estonia, Argentina Korea, Mexico, Tunisia, Sri Lanka, Pakistan, India, Thailand, Turkey, Indonesia, South Africa, Philippines |
| Thailand | Greece, Slovenia | Croatia, Kuwait | Jordan | Morocco, China, Bangladesh, Russia, Lebanon, | Brazil, Venezuela, Oman, Hungary, Romania, Kenya, Argentina, Egypt, Lithuania, Colombia, Chile, Poland, Estonia, Korea, Pakistan, Tunisia, Mexico, Sri Lanka, Philippines, India, Indonesia, South Africa, Turkey, Thailand |
| Egypt | Greece | Malaysia, Slovenia, Bangladesh | - | Venezuela, Argentina | Philippines, China, Sri Lanka, Croatia, Colombia, Kuwait, Pakistan, Morocco, Chile, Jordan, Tunisia, Indonesia, Brazil, Thailand, Mexico, Russia, South Africa, Turkey, Estonia, India, Hungary, Korea, Lebanon, Romania, Lithuania, Kenya, Poland, Oman |
| Greece | Tunisia, Indonesia, Thailand, Sri Lanka, Columbia, South Africa, Mexico, Bangladesh, Chile, Turkey, Argentine, Philippine, Pakistan, India, Korea, Venezuela, Brazil, Estonia, Poland, Egypt, Lithuania, Oman | Morocco, China, Romania, Russia, Jordan | - | Kuwait, Croatia, Malaysia | Slovenia, Greece |
| Jordan | Bangladesh, Malaysia | Philippines, Argentina, Venezuela, Sri Lanka, Greece, Pakistan, Indonesia | Thailand, Tunisia, China, South Africa | India, Turkey, Slovenia, Mexico, Chile, Korea, Colombia, Morocco, Estonia | Poland, Brazil, Lithuania, Hungary, Kenya, Romania, Egypt, Croatia, Russia, Oman, Lebanon, Kuwait |
| Kuwait | Greece | Bangladesh, Argentina, China, Venezuela, Philippines, Croatia, Pakistan | Morocco, Thailand, Sri Lanka, Jordan, Indonesia | South Africa, Lithuania, India, Romania, Estonia | Tunisia, Turkey, Russia, Oman, Kenya, Hungary, Egypt, Poland, Lebanon, Korea, Mexico, Colombia, Chile |
| Lebanon | - | Malaysia, Argentine | Sri Lanka | Venezuela, Tunisia, Indonesia, Pakistan, Thailand, South Africa, Turkey | India, Mexico, Colombia, Chile, Greece, Korea, China, Morocco, Brazil, Estonia, Poland, Slovenia, Egypt, Lithuania, Kuwait, Hungary, Kenya Oman, Russia, Romania, Croatia, Jordan |
| Oman | Greece | Slovenia, Bangladesh | Argentina | Venezuela, Philippines, Pakistan, Thailand | Sri Lanka, China, Indonesia, South Africa, Estonia, Croatia, Tunisia, Mexico, India, Turkey, Colombia, Kuwait, Lithuania, Chile, Romania, Korea, Brazil, Morocco, Poland, Kenya, Hungary, Jordan, Russia, Egypt, Lebanon |

**Table S2 cont’d** International equity portfolios for individual emerging market by correlations

| Portfolios | Yellow | Blue | Purple | Green | Red |
| --- | --- | --- | --- | --- | --- |
| Correlation range | -100% - 0.1% | 0% - 20% | 21% - 39% | 40% - 59% | 60% - 100% |
| Kenya | - | Slovenia, Malaysia, Bangladesh |  | Argentina, Philippines, Venezuela, Pakistan | China, Mexico, Indonesia, India, South Africa, Lebanon, Egypt, Tunisia, Sri Lanka, Chile, Colombia, Lithuania, Oman, Thailand, Croatia, Morocco, Russia, Poland, Kuwait, Romania, Brazil, Hungary, Turkey, Korea, Estonia, Jordan |
| Morocco | - | Greece, Malaysia, Argentina, Slovenia, | Venezuela, Pakistan | Bangladesh, Thailand, Estonia, Kuwait, Romania, Lithuania, Philippine, Sri Lanka, Colombia, Mexico, Jordan, Indonesia, South Africa, Turkey | Croatia, Chile, Hungary, Brazil, India, Kenya, Tunisia, Poland, Korea, Russia, Egypt, China, Oman, Lebanon |
| Tunisia | Greece, Slovenia, Malaysia | Kuwait, Croatia | Jordan | Russia, China, Romania | Venezuela, Bangladesh, Kenya, Lithuania, Hungary Morocco, Argentina, Oman, Lebanon, Brazil, Estonia, Egypt, Poland, Pakistan, Colombia, Philippine, Thailand, Chile, Mexico, Sri Lanka, Korea, India, South Africa, Indonesia, Turkey, Tunisia |
| Hungary | - | Malaysia, Bangladesh, Slovenia | - | Venezuela, Philippine, Argentina | Morocco, Sri Lanka, Colombia, China, Thailand, Tunisia, Indonesia, Pakistan, Chile, South Africa, Jordan, Croatia, Mexico, Kuwait, Brazil, India, Turkey, Oman, Russia, Korea Lebanon, Kenya, Egypt, Poland, Estonia, Romania, Lithuania, Hungary |
| Poland | Greece | Slovenia, Malaysia, Bangladesh | - | Argentina, Venezuela | Jordan, Kuwait, Morocco, China, Croatia, Philippines, Colombia, Pakistan, Sri Lanka, Tunisia, Chile, Brazil, Indonesia, Lebanon, Thailand, Mexico, Russia, Oman, South Africa, India , Turkey, Hungary, Korea, Egypt, Lithuania, Romania, Kenya, Estonia, Poland |
| Russia | - | Greece, Argentina, Bangladesh | Philippines, Pakistan, Venezuela, Slovenia | Sri Lanka, Thailand Indonesia, South Africa, Tunisia | China, India, Mexico, Turkey, Colombia, Chile, Morocco, Estonia, Brazil, Lithuania, Korea, Jordan, Egypt, Kuwait, Hungary, Romania, Kenya, Croatia, Poland, Lebanon, Oman |
| South Africa | Greece, Slovenia | Malaysia | Kuwait, Croatia, Jordan | Bangladesh, Russia, Morocco | China, Lebanon, Venezuela, Brazil, Oman, Romania Hungary, Kenya, Colombia, Chile, Lithuania, Argentina, Egypt, Poland, Sri Lanka, Estonia, Korea, Mexico, Tunisia, Philippines, Pakistan, Thailand, Indonesia, India, Turkey |
| Turkey | Greece, Slovenia | Malaysia | Kuwait, Croatia | Jordan, Bangladesh, China, Morocco | Russia, Venezuela, Lebanon, Oman, Brazil, Argentina, Romania, Hungary, Kenya, Colombia, Lithuania, Egypt, Pakistan, Chile, Poland, Estonia, Philippines, Sri Lanka, Mexico, Tunisia, Korea, Indonesia, India, Thailand, South Africa |
| Croatia | Bangladesh | Argentina, Philippines, Sri Lanka, Venezuela, Tunisia, Thailand,, Pakistan, Malaysia, Indonesia, Colombia | Mexico, South Africa, Chile, Turkey, India | Greece, Korea, Brazil | Estonia, Morocco, China, Lebanon, Lithuania, Egypt, Poland, Kenya, Oman, Hungary, Jordan, Slovenia, Romania, Russia, Kuwait |
| Estonia | Greece | Slovenia, Malaysia, Bangladesh | - | Morocco, China, Jordan | Kuwait, Croatia, Venezuela, Lebanon, Argentine, Colombia, Russia, Brazil, Tunisia, Oman, Philippine, Chile, Sri Lanka, Indonesia, Pakistan, Mexico, Egypt, India, Thailand, Korea, Kenya, South Africa, Turkey, Hungary, Romania, Poland, Lithuania, Estonia |
| Lithuania | Greece | Bangladesh, Malaysia, Slovenia | - | Morocco, Venezuela, China | Kuwait, Croatia, Lebanon, Argentine, Colombia, Russia, Brazil, Tunisia, Oman, Philippine, Chile, Jordan, Sri Lanka, Indonesia, Pakistan, Mexico, Egypt, India, Thailand, Korea, Kenya, South Africa, Turkey, Hungary, Romania, Poland, Lithuania, Estonia |
| Romania | - | Greece, Malaysia | Slovenia | Venezuela, Argentine, Morocco, Philippine, Sri Lanka, Tunisia | Kuwait, Croatia, Lebanon, China, Colombia, Russia, Brazil, Oman, Chile, Jordan, Indonesia, Pakistan, Mexico, Egypt, India, Thailand, Korea, Kenya, South Africa, Turkey, Hungary, Romania, Poland, Lithuania, Estonia |
| Slovenia | Sri Lanka, Bangladesh, Tunisia, Philippine, Thailand, Indonesia, Columbia, Mexico, Argentine, Chile, Turkey, South Africa, Venezuela, Pakistan, India, Korea, Brazil | Estonia, Poland, Lebanon, Lithuania, Egypt, Hungary, Kenya, Morocco, Oman | Romania, Malaysia, Russia | Jordan, China | Kuwait, Croatia, Greece, Slovenia |

**Table S3** Descriptive Statistics

| Countries | Mean | Std. deviation | Skewness | Kurtosis | Mean | Std. deviation | Skewness | Kurtosis |
| --- | --- | --- | --- | --- | --- | --- | --- | --- |
|  | Price Statistics | | | | Return Statistics | | | |
| ***Latin American*** | | | | | | | | |
| Argentina | 3524.1972 | 4021.7715 | 1.8572 | 2.4395 | 0.0015 | 0.0222 | -0.2225 | 4.1885 |
| Brazil | 43115.3142 | 18813.6686 | -0.4455 | -1.1505 | 0.0008 | 0.0185 | -0.1264 | 4.2236 |
| Chile | 2948.5305 | 1214.5272 | -0.2356 | -1.3344 | 0.0009 | 0.0106 | -0.0058 | 9.5015 |
| Colombia | 3117.8752 | 1629.6753 | -0.3043 | -1.1588 | 0.0016 | 0.0133 | -0.0959 | 20.8092 |
| Mexico | 27168.2456 | 13941.3646 | -0.2302 | -1.3959 | 0.0015 | 0.0122 | 0.0536 | 6.0103 |
| Venezuela | 81.0514 | 88.3705 | 2.6575 | 8.5476 | 0.0018 | 0.0655 | -12.7145 | 694.8166 |
| ***Asian*** | | | | | | | | |
| Bangladesh | 9051.9325 | 4530.1289 | -0.0354 | -0.8753 | 0.0025 | 0.0132 | 1.2501 | 12.5435 |
| China | 2332.2432 | 912.9756 | 1.2145 | 1.7366 | 0.0001 | 0.0165 | -0.3102 | 4.8244 |
| India | 13802.0996 | 8075.7017 | 0.1847 | -1.1712 | 0.0004 | 0.0151 | -0.1963 | 7.5908 |
| Indonesia | 2479.5306 | 1733.1534 | 0.2739 | -1.4499 | 0.0003 | 0.0143 | -0.6344 | 5.8832 |
| Korea | 1430.8505 | 540.8231 | -0.3296 | -1.4628 | 0.0006 | 0.0152 | -0.6375 | 6.5825 |
| Malaysia | 2566.0728 | 507.8952 | 0.5035 | -0.2147 | 0.0005 | 0.0135 | -0.1012 | 5.3264 |
| Pakistan | 13202.9825 | 10959.1681 | 1.0708 | 0.1585 | 0.0017 | 0.0134 | -0.3982 | 3.4079 |
| Philippines | 329.3284 | 188.5613 | 0.4164 | -1.1904 | 0.0004 | 0.0134 | -0.4003 | 6.1381 |
| Sri Lanka | 3566.8403 | 2426.0605 | 0.2568 | -1.5255 | 0.0012 | 0.0116 | -0.5085 | 19.8101 |
| Thailand | 848.1995 | 406.6356 | 0.3848 | -1.1075 | 0.0008 | 0.0131 | -0.7316 | 10.1932 |
| ***MENA*** | | | | | | | | |
| Egypt | 464.0842 | 274.0895 | -0.1965 | -1.1052 | 0.0004 | 0.0165 | -1.5081 | 25.2999 |
| Greece | 2251.8153 | 1342.6778 | 0.5726 | -0.7213 | -0.0005 | 0.0187 | -0.3330 | 6.8613 |
| Jordan | 4432.7216 | 2049.7469 | 0.3749 | -0.0746 | 0.0002 | 0.0108 | -0.3577 | 60.2479 |
| Kenya | 3560.3625 | 1268.5676 | -0.3365 | -0.9425 | 0.0001 | 0.0137 | -0.2170 | 732.5383 |
| Kuwait | 434.8512 | 187.4297 | 0.6468 | 0.2655 | 0.0002 | 0.0115 | 2.6694 | 152.4685 |
| Lebanon | 1044.3301 | 399.1684 | -0.1815 | -0.8708 | 0.0001 | 0.0125 | -0.9094 | 189.4453 |
| Morocco | 8767.6054 | 3240.4243 | -0.4222 | -0.9644 | 0.0003 | 0.0075 | -0.5615 | 7.7322 |
| Oman | 5077.3712 | 2183.9145 | 0.1756 | 0.1767 | 0.0002 | 0.0098 | -0.5518 | 22.7195 |
| Tunisia | 3136.7906 | 1634.7602 | 0.0883 | -1.6601 | 0.0003 | 0.0053 | -0.1602 | 12.3058 |
| ***CEE*** | | | | | | | | |
| Croatia | 1928.5892 | 950.8928 | 1.7198 | 2.8703 | 0.0002 | 0.0129 | 0.0763 | 19.9496 |
| Estonia | 553.5655 | 272.8817 | -0.1647 | -1.2566 | 0.0004 | 0.0109 | 0.1260 | 9.4844 |
| Hungary | 17208.7399 | 6462.2341 | -0.2261 | -1.10852 | 0.0003 | 0.0151 | -0.1206 | 6.4223 |
| Lithuania | 321.9154 | 154.0115 | -0.3284 | -1.2675 | 0.0004 | 0.0106 | -0.5439 | 28.4157 |
| Poland | 36566.4718 | 14414.7985 | -0.2385 | -1.2146 | 0.0002 | 0.0126 | -0.3677 | 3.6939 |
| Romania | 4768.2601 | 2569.0695 | -0.2035 | -0.9368 | 0.0006 | 0.0156 | -0.4017 | 9.3561 |
| Russia | 1057.4125 | 588.6224 | 0.1624 | -1.0059 | 0.0004 | 0.0219 | -0.3942 | 8.5790 |
| Slovenia | 1005.3642 | 516.6086 | 1.7085 | 2.0157 | -0.0001 | 0.0156 | -0.1132 | 323.5769 |
| South Africa | 26517.3096 | 14997.2566 | 0.3696 | -1.1015 | 0.0004 | 0.0120 | -0.1757 | 3.7160 |
| Turkey | 44461.6543 | 25421.6599 | 0.0705 | -1.3852 | 0.0003 | 0.0214 | -0.0615 | 7.7453 |

**Table S4-1** Panel Cointegration tests: Latin America

|  |  | Kao Panel Cointegration | Pedroni Panel Co-integration statistics | | | | | | | Johansen Panel Co-integration  Trace statistics | | | |
| --- | --- | --- | --- | --- | --- | --- | --- | --- | --- | --- | --- | --- | --- |
|  |  | ADF t-Stat. | Panel v | Panel rho | Panel PP | Panel ADF | Group rho | Group PP | Group ADF | None | 1 | 2 | 3 |
| ***Model 1*** |  |  |  |  |  |  |  |  |  |  |  |  |  |
| Blue |  | -2.4267* | -1.5915 | 0.6163 | 0.8920 | -0.0244 | 2.2499 | 2.2837 | 1.1958 | 5.537 | 3.804 | - | - |
| Purple |  | 1.3926** | -2.6846 | 2.7881 | 3.3514 | 3.0342 | 0.1601 | 2.8449 | 2.609 | 22.32 | 31.28** | - | - |
| Green |  | 0.6313 | -1.5894 | 3.1302 | 4.4115 | 2.6337 | -0.4738 | 5.3265 | 4.2679 | 143.6* | 138.5* | - | - |
| Red |  | 0.9476 | 3.7310* | -0.5086 | 2.3956 | 0.6969 | 0.0175 | 5.8993 | 3.5264 | 287.8* | 312.2* | - | - |
| ***Model 2*** |  |  |  |  |  |  |  |  |  |  |  |  |  |
| Blue |  | -1.5769 | -0.7535 | -0.2678 | 0.5905 | 0.7272 | 1.0148 | 2.1376 | 2.1285 | 8.267 | 5.485 | 13.66 | - |
| Purple |  | 0.6522 | -2.4376 | 2.112 | 2.5137 | 2.9822 | -0.6842 | 0.7652 | 1.4836 | 15.37 | 9.392 | 22.11 | - |
| Green |  | 0.2613 | -2.2898 | 4.1295 | 5.9815 | 4.8475 | -1.079 | 2.8318 | 3.3625 | 83.36 | 36.88 | 78.29 | - |
| Red |  | 0.5254 | 2.4507 | 0.6866 | 3.6666 | 1.5315 | -0.8121 | 4.6118 | 1.8842 | 286.1* | 136.7 | 256.5 | - |
| ***Model 3*** |  |  |  |  |  |  |  |  |  |  |  |  |  |
| Blue |  | -1.5160** | 1.5776* | -7.8432* | -2.4800* | -6.7149* | -16.2683* | -7.0372* | -9.0580* | 385.5* | 22.77 | 10.51 | 37.83 |
| Purple |  | -1.4099** | -2.518 | -5.9847* | -2.6713* | -4.6567* | -6.8459* | -3.7595* | -4.9798* | 232.2* | 12.52 | 7.75 | 18.12 |
| Green |  | -1.8566* | -1.406 | -12.8702* | -4.6901* | -7.6121* | -26.7960* | -10.0334* | -9.1478* | 863.6* | 84.33 | 30.61 | 73.69 |
| Red |  | -7.6479* | 0.6582 | -2.6008* | 3.4198* | 0.4138* | -21.5433* | -6.0177* | -9.6714* | 2641* | 269.9*** | 143.9 | 307.4* |
| ***Model 4*** |  |  |  |  |  |  |  |  |  |  |  |  |  |
| Blue |  | -1.0537 | -0.9472 | 4.5040 | 5.5151 | 4.9196 | 0.6217 | 2.6317 | 3.1201 | 622* | 43.83 | 13.47 | 43.1 |
| Purple |  | 0.5947 | -2.9143 | 2.3053 | 2.6697 | 4.0563 | 0.1715 | 1.1658 | 2.8336 | 202.9* | 14.66 | 8.584 | 23.56 |
| Green |  | 0.2215 | -3.6917 | 5.5252 | 7.7589 | 7.4092 | 1.1753 | 4.5410 | 6.2644 | 824.5* | 79.42 | 34.74 | 78.63 |
| Red |  | 0.3600 | -0.7867 | 3.9263 | 6.8825 | 5.5464 | 0.9803 | 5.5053 | 5.8657 | 2523* | 270.2** | 128.6 | 249.2 |
| ***Model 5*** |  |  |  |  |  |  |  |  |  |  |  |  |  |
| Blue |  | -2.9484* | -0.5976 | 2.1357 | 2.6925 | 2.9063 | 3.2658 | 3.9929 | 3.7841 | 453.5* | 45.66 | 23.78 | 38.76 |
| Purple |  | -1.5618* | 1.1589 | 0.9638 | 1.2843 | 2.5573 | 1.5124 | 2.2627 | 3.6314 | 215.7* | 24.29 | 14.43 | 19.49 |
| Green |  | -2.5721* | 1.9269 | 0.3738 | 1.6403 | 1.9198 | 3.3283 | 5.4101 | 5.5372 | 741.1* | 113.5 | 66.89 | 99.25 |
| Red |  | -7.7347* | 2.7842 | -2.1867 | -1.4036 | -1.0098 | -0.2736 | 1.0937 | -0.3166 | 2476.0* | 549.7* | 339.9* | 537.9* |

Note: This table presents results from three cointegration tests, namely Kao (1999), Maddala and Wu (1999), and Pedroni (1999, 2004). The Pedroni and Kao are single-equation tests while the Maddala and Wu cointegration test uses a system of equations. They all test the null of no cointegration. To examine the stationarity of the residuals, Kao uses the standard ADF test while Pedroni uses a variety of tests, including the ADF test. For the Maddala and Wu test, we report the trace test to indicate the number of cointegration relations among variables of a system. Relationships in five models (1-5) (in column 1) were tested region-wise, after incorporating portfolios developed under the high-low correlation strategy. Results are displayed by portfolios, which are based on the correlation between emerging markets *i* from Latin American nations and *j* markets from Asia, CEE, MENA, and the rest of Latin America. Blue portfolio captures correlation of 0.2 or lower between these markets; purple portfolio with a correlation between 0.2 and 0.3; green portfolio with a correlation between 0.4 and 0.5; and red portfolio stock having a correlation of 0.6 or more. * denotes rejection of the null hypothesis of no cointegration at 5% or better.

**Table S4-2** Panel Co-Integration Tests: Asia

|  |  | Kao Panel Cointegration | Pedroni Panel Co-integration statistics | | | | | | | Johansen Panel Co-integration  Trace statistics | | | |
| --- | --- | --- | --- | --- | --- | --- | --- | --- | --- | --- | --- | --- | --- |
|  |  | ADF t-Stat. | Panel v | Panel rho | Panel PP | Panel ADF | Group rho | Group PP | Group ADF | None | 1 | 2 | 3 |
| ***Model 1*** |  |  |  |  |  |  |  |  |  |  |  |  |  |
| Blue |  | 15.4364* | -5.1323 | 7.7016 | 15.1512 | 14.9767 | 3.8980 | 4.4767 | 4.8613 | 55.93 | 87.78 | - | - |
| Purple |  | 2.0186* | -4.8876 | 10.0356 | 21.5930 | 19.5941 | 7.2181 | 14.0095 | 11.3298 | 112.6* | 98.87* | - | - |
| Green |  | 25.3374 | -5.3825 | 13.3843 | 26.2862 | 23.5202 | 10.1649 | 17.6436 | 14.0126 | 118.7 | 106.8 | - | - |
| Red |  | 30.2368* | -5.7894 | 11.5916 | 19.7623 | 19.6400 | 5.6723 | 15.6293 | 14.4321 | 547.4* | 332.9 | - | - |
| ***Model 2*** |  |  |  |  |  |  |  |  |  |  |  |  |  |
| Blue |  | 13.3518* | 1.4874 | -3.3758 | -1.2263 | -2.6686 | 2.3660 | 2.4811 | 1.6230 | 60.22 | 27.46 | 48.77 | - |
| Purple |  | 13.1149* | -3.1026 | 2.4441 | 5.7972 | 0.4908 | 5.7345 | 8.1368 | 3.9322 | 5.7345 | 8.1368 | 3.9322 | - |
| Green |  | 20.0413* | -1.5052 | 2.0966 | 7.5716 | 3.3880 | 6.5636 | 9.0843 | 5.8615 | 88.00 | 50.62 | 108.7 | - |
| Red |  | 15.4082* | 1.7201* | -1.3242* | 6.9855 | 2.8810 | 0.8294 | 6.0427 | 3.0123 | 707.9 | 278.2 | 401.8 | - |
| ***Model 3*** |  |  |  |  |  |  |  |  |  |  |  |  |  |
| Blue |  | 11.4089* | 1.4881* | -18.9780* | -9.6380* | -9.6458* | -18.068* | -6.9932* | -6.3147* | 914.1* | 50.67 | 24.74 | 49.20 |
| Purple |  | 7.3553* | -3.9464 | -5.0207* | 0.4860 | -1.5307* | -10.4633* | -2.9234* | -1.5307* | 800.0* | 51.45 | 28.72 | 43.46 |
| Green |  | 14.1720* | -2.9409 | -5.3072* | 0.5586* | 1.1184* | -16.7146* | -3.3976* | -0.9692 | 1352.0* | 57.99 | 36.78 | 99.89 |
| Red |  | 6.5069* | -0.4599 | -11.5694* | -2.8458* | -8.3515* | -49.6159* | -17.7169* | -20.4069* | 5058* | 460.9* | 236.6 | 326.4 |
| ***Model 4*** |  |  |  |  |  |  |  |  |  |  |  |  |  |
| Blue |  | 13.3541* | -0.3394 | -1.3467 | 0.3173 | -0.5232 | 3.9992 | 4.5703 | 4.5271 | 1148.0* | 60.70 | 27.50 | 49.84 |
| Purple |  | 13.1399* | -4.2525 | 4.2511 | 7.9829 | 2.9799 | 6.3549 | 9.6140 | 6.9880 | 836.0* | 81.34 | 41.58 | 54.55 |
| Green |  | 1.5541* | -2.7188 | 4.4270 | 7.3964 | 8.6866 | 9.0349 | 12.5686 | 13.4970 | 1091.0* | 86.27 | 50.73 | 112.6 |
| Red |  | 15.3880* | -2.0559 | 2.9004 | 11.1229 | 8.1027 | 3.1727 | 8.7051 | 8.3020 | 4107.0* | 726.3* | 276.1 | 404.7* |
| ***Model 5*** |  |  |  |  |  |  |  |  |  |  |  |  |  |
| Blue |  | 13.3474* | -30.5847 | -0.2915 | 1.6371 | 0.2948 | 4.8132 | 5.3606 | 4.4651 | 492.3* | 59.48 | 25.64 | 45.53 |
| Purple |  | 12.8827* | -4.2597 | 4.4357 | 8.3279 | 2.9955 | 7.7731 | 11.2331 | 7.0337 | 520.4* | 71.60 | 30.26 | 49.39 |
| Green |  | 18.8176* | -3.4044 | 5.1036 | 11.2522 | 7.1659 | 7.4031 | 11.8113 | 7.7393 | 749.9* | 93.47 | 50.77 | 119.4 |
| Red |  | 6.7211* | -1.8881 | 4.3058 | 13.1799 | 8.6916 | -5.7755* | 3.9364 | 1.3982 | 3428.0* | 871.5* | 315.2 | 459.2* |

Note: This table presents results from three cointegration tests, namely Kao (1999), Maddala and Wu (1999), and Pedroni (1999, 2004). The Pedroni and Kao are single-equation tests while the Maddala and Wu cointegration test uses a system of equations. They all test the null of no cointegration. To examine the stationarity of the residuals, Kao uses the standard ADF test while Pedroni uses a variety of tests, including the ADF test. For the Maddala and Wu test, we report the trace test to indicate the number of cointegration relations among variables of a system. Relationships in five models (1-5) (in column 1) were tested region-wise, after incorporating portfolios developed under the high-low correlation strategy. Results are displayed by portfolios, which are based on the correlation between emerging markets *i* from Asian nations and *j* markets from CEE, Latin American, MENA, and the rest of Asia. Blue portfolio captures correlation of 0.2 or lower between these markets; purple portfolio with a correlation between 0.2 and 0.3; green portfolio with a correlation between 0.4 and 0.5; and red portfolio stock having a correlation of 0.6 or more. * denotes rejection of the null hypothesis of no cointegration at 5% or better.

**Table S4-3** Panel Co-Integration Tests: MENA

|  | | |  | Kao Panel Cointegration | | Pedroni Panel Co-integration statistics | | | | | | | | | | | | | | Johansen Panel Co-integration  Trace statistics | | | | | | | |
| --- | --- | --- | --- | --- | --- | --- | --- | --- | --- | --- | --- | --- | --- | --- | --- | --- | --- | --- | --- | --- | --- | --- | --- | --- | --- | --- | --- |
|  | | |  | ADF t-Stat. | | Panel v | | Panel rho | | Panel PP | | Panel ADF | | Group rho | | Group PP | | Group ADF | | None | | 1 | | 2 | | 3 | |
| ***Model 1*** | | |  |  | |  | |  | |  | |  | |  | |  | |  | |  | |  | |  | |  | |
| Blue | | |  | -2.8973* | | -3.1155 | | 2.6769 | | 0.9141 | | 1.0336 | | 3.9424 | | 1.6590 | | 2.4646 | | 92.66* | | 116.3* | | - | | - | |
| Purple | | |  | -2.6081* | | -2.8812 | | 2.9222 | | 2.1213 | | 1.7790 | | 4.5619 | | 3.6573 | | 3.6573 | | 128.6* | | 115.1* | | - | | - | |
| Green | | |  | -3.4243* | | -3.6978 | | 3.7675 | | 3.6027 | | 2.6077 | | 6.1261 | | 5.8306 | | 5.1866 | | 153.8* | | 152.3* | | - | | - | |
| Red | | |  | -4.6534* | | -1.0662 | | 0.5510 | | 1.6456 | | 0.1540 | | 4.1951 | | 4.7640 | | 2.8389 | | 576.2* | | 546.3* | | - | | - | |
| ***Model 2*** | | |  |  | |  | |  | |  | |  | |  | |  | |  | |  | |  | |  | |  | |
| Blue | | |  | -2.7233* | | -3.7917 | | 3.3217 | | 1.1772 | | 1.6297 | | 5.0088 | | 1.8003 | | 2.7936 | | 40.50 | | 11.74 | | 26.40 | | - | |
| Purple | | |  | -2.6994* | | -3.8016 | | 4.6844 | | 5.0445 | | 4.5052 | | 6.3891 | | 6.2751 | | 6.0827 | | 78.84 | | 29.47 | | 46.75 | | - | |
| Green | | |  | -3.4935* | | -4.6130 | | 4.8925 | | 5.2497 | | 4.4449 | | 6.9152 | | 6.9735 | | 6.2770 | | 121.9* | | 49.60* | | 75.68 | | - | |
| Red | | |  | -5.3563* | | -3.0929 | | 3.0541 | | 3.8751 | | 2.2865 | | 5.6088 | | 6.0446 | | 3.7565 | | 334.2* | | 83.56 | | 195.0 | | - | |
| ***Model 3*** | | |  |  | |  | |  | |  | |  | |  | |  | |  | |  | |  | |  | |  | |
| Blue | | |  | -6.4097* | | -3.6301 | | -36.8573* | | -20.3549* | | 12.0348* | | -41.5107* | | -16.8590* | | -9.9912* | | 862.3* | | 29.93 | | 5.552 | | 27.87 | |
| Purple | | |  | -12.9033* | | -3.1099 | | -42.5850* | | -21.4205* | | -15.9823* | | -40.4270* | | -20.1994* | | -14.3309* | | 990.9* | | 57.78 | | 21.14 | | 44.66 | |
| Green | | |  | -16.8547* | | -4.4940 | | -35.2936* | | -19.3149* | | -15.1250* | | -48.2978* | | -19.2357* | | -17.1435* | | 1592.0* | | 98.38 | | 46.20 | | 68.42 | |
| Red | | |  | -33.2299* | | -2.8684 | | -93.4611* | | -48.2433* | | -29.6239* | | -86.3282* | | -35.7601* | | -30.5006* | | 5942.0* | | 336.9* | | 88.72 | | 251.5 | |
| ***Model 4*** | | |  |  | |  | |  | |  | |  | |  | |  | |  | |  | |  | |  | |  | |
| Blue | | |  | -2.7288* | | -4.6911 | | 4.5844 | | 3.2959 | | 4.0294 | | 5.9964 | | 3.7799 | | 5.1364 | | 789.9* | | 39.58 | | 11.41 | | 26.56 | |
| Purple | | |  | -2.7631* | | -4.7864 | | 5.9209 | | 7.0036 | | 7.1302 | | 7.6811 | | 8.3565 | | 8.8284 | | 823.8* | | 77.93 | | 29.42 | | 47.14 | |
| Green | | |  | -3.6061* | | -5.6783 | | 4.9515 | | 5.3654 | | 6.6871 | | 7.5808 | | 8.1328 | | 9.1065 | | 1264.0* | | 120.6* | | 48.81 | | 76.55 | |
| Red | | |  | -5.3860* | | -5.9077 | | 5.5491 | | 6.5843 | | 6.8090 | | 8.5603 | | 9.5793 | | 9.1854 | | 4092.0* | | 324.3* | | 82.20 | | 196.2 | |
| ***Model 5*** | | |  |  | |  | |  | |  | |  | |  | |  | |  | |  | |  | |  | |  | |
| Blue |  | 0.5563 | | | -5.2716 | | 4.6882 | | 4.4776 | | 4.8016 | | 7.0216 | | 6.9780 | | 7.5306 | | 426.5* | | 62.60 | | 28.46 | | 42.88 | |  |
| Purple |  | -2.2889* | | | -4.3476 | | 5.5038 | | 7.6538 | | 6.7451 | | 7.7949 | | 9.9681 | | 9.7199 | | 488.4* | | 58.53 | | 24.03 | | 42.59 | |  |
| Green |  | -3.5715* | | | -5.1721 | | 5.1584 | | 6.8925 | | 5.7660 | | 6.7766 | | 9.1718 | | 8.3196 | | 666.4* | | 112.7 | | 46.00 | | 72.48 | |  |
| Red |  | -6.4778* | | | -4.2675 | | 3.7557 | | 6.4705 | | 4.2767 | | 6.6070 | | 9.6895 | | 7.4101 | | 2677.0* | | 276.5 | | 130.6 | | 257.2 | |  |

Note: This table presents results from three cointegration tests, namely Kao (1999), Maddala and Wu (1999), and Pedroni (1999, 2004). The Pedroni and Kao are single-equation tests while the Maddala and Wu cointegration test uses a system of equations. They all test the null of no cointegration. To examine the stationarity of the residuals, Kao uses the standard ADF test while Pedroni uses a variety of tests, including the ADF test. For the Maddala and Wu test, we report the trace test to indicate the number of cointegration relations among variables of a system. Relationships in five models (1-5) (in column 1) were tested region-wise, after incorporating portfolios developed under the high-low correlation strategies. Results are displayed by portfolios, which are based on the correlation between emerging markets *i* from MENA nations and *j* markets from Asia, CEE, Latin America, and the rest of MENA. Blue portfolio captures correlation of 0.2 or lower between these markets; purple portfolio with a correlation between 0.2 and 0.3; green portfolio with a correlation between 0.4 and 0.5; and red portfolio stock having a correlation of 0.6 or more. * denotes rejection of the null hypothesis of no cointegration at 5% or better.

**Table S4-4** Panel Cointegration tests: CEE

|  |  | Kao Panel Cointegration | Pedroni Panel Co-integration statistics | | | | | | | Johansen Panel Co-integration  Trace statistics | | | |
| --- | --- | --- | --- | --- | --- | --- | --- | --- | --- | --- | --- | --- | --- |
|  |  | ADF t-Stat. | Panel v | Panel rho | Panel PP | Panel ADF | Group rho | Group PP | Group ADF | None | 1 | 2 | 3 |
| ***Model 1*** |  |  |  |  |  |  |  |  |  |  |  |  |  |
| Blue |  | 2.1236* | -2.7931 | 3.2561 | 3.4578 | 3.5545 | 6.2599 | 6.7729 | 7.4152 | 83.80 | 105.4* | - | - |
| Purple |  | 3.4881* | -0.2668 | 1.6569 | 1.3904 | 6.3581 | 4.8704 | 5.1126 | 6.8357 | 108.2* | 83.22* | - | - |
| Green |  | 1.2274 | -1.7296 | 2.4352 | 3.1823 | 3.5746 | 4.3511 | 4.5996 | 4.7267 | 99.00* | 79.69 | - | - |
| Red |  | 5.7764* | 3.0204* | -0.1732 | 2.7866 | 1.5376 | 4.4456 | 7.1946 | 5.6197 | 580.3* | 519.0 | - | - |
| ***Model 2*** |  |  |  |  |  |  |  |  |  |  |  |  |  |
| Blue |  | 0.9913 | -0.0512 | 2.4326 | 2.6648 | 1.9872 | 6.2389 | 5.9846 | 6.1900 | 81.14 | 28.63 | 49.58 | - |
| Purple |  | 2.1146* | 5.0752* | -3.4131* | -4.0585* | -0.8606 | 2.3419 | 1.9902 | 3.3811 | 105.4* | 31.07 | 58.66 | - |
| Green |  | -0.2924 | 0.1867 | 1.2025 | 1.0622 | 0.5201 | 4.4472 | 3.3013 | 2.9076 | 69.66* | 28.15 | 58.73 | - |
| Red |  | 0.4596 | 4.1424* | -0.9155 | -0.0333 | -2.2938* | 3.1864 | 3.5859 | 1.3940 | 440.1 | 150.9 | 339.7 | - |
| ***Model 3*** |  |  |  |  |  |  |  |  |  |  |  |  |  |
| Blue |  | -3.5115* | 2.3748* | -84.9826* | -36.7218* | -23.8804* | -60.7163* | -19.8983* | -10.7901* | 972.1* | 60.49 | 23.63 | 47.46 |
| Purple |  | -5.0186* | -0.0203 | -57.2178* | -27.0735* | -20.5236* | -37.3233* | -11.9901* | -7.8749* | 717.6* | 73.29* | 28.17 | 50.57 |
| Green |  | -4.2327* | 1.3548* | -28.8223* | -15.7873* | -15.7191* | -53.6505* | -17.9785* | -11.9925* | 986.4* | 62.33 | 27.29 | 52.26 |
| Red |  | -16.4889* | 5.2240* | -55.8195* | -32.8540* | -33.6598* | -76.9342* | -28.1051* | -25.1460* | 7515.0* | 439.3 | 182.8 | 377.8 |
| ***Model 4*** |  |  |  |  |  |  |  |  |  |  |  |  |  |
| Blue |  | 0.9960 | -1.7335 | 4.2751 | 4.7617 | 4.4417 | 6.5311 | 6.0168 | 8.4294 | 983.6* | 73.65 | 28.04 | 53.24 |
| Purple |  | 2.1262* | 3.2025* | -2.9516* | -3.8835* | 0.7577 | 1.7556 | 1.0665 | 4.7637 | 673.0* | 101.5* | 30.36 | 60.80 |
| Green |  | -0.2844 | -1.1933 | 1.2962 | 1.4035 | 2.6172 | 5.7609 | 5.1337 | 5.5301 | 739.2* | 67.33 | 27.11 | 59.17 |
| Red |  | 0.4803 | -0.2275 | 2.6948 | 3.4538 | 2.9068 | 6.9030 | 7.6642 | 7.5768 | 5772.0* | 430.8 | 150.3 | 342.8 |
| ***Model 5*** |  |  |  |  |  |  |  |  |  |  |  |  |  |
| Blue |  | 0.5999 | -1.5634 | 2.9940 | 3.2771 | 3.2891 | 7.5664 | 8.7838 | 9.4526 | 438.2* | 53.86 | 32.06 | 49.32 |
| Purple |  | 1.8290* | 3.0976* | -1.3745* | -2.5755* | 0.9744 | 3.4259 | 3.8128 | 5.1390 | 376.8* | 87.73* | 32.02 | 44.21 |
| Green |  | -0.2740 | -1.2329 | 2.8924 | 3.0427 | 2.4688 | 6.0261 | 6.3182 | 5.8136 | 517.2* | 79.56 | 41.17 | 72.58 |
| Red |  | 0.0473 | 1.1612 | 2.7131 | 3.5066 | 1.4357 | 5.8982 | 7.3459 | 5.4373 | 2978.0* | 466.3 | 240.6 | 433.1 |

Note: This table presents results from three cointegration tests, namely Kao (1999), Maddala and Wu (1999), and Pedroni (1999, 2004). The Pedroni and Kao are single-equation tests while the Maddala and Wu cointegration test uses a system of equations. They all test the null of no cointegration. To examine the stationarity of the residuals, Kao uses the standard ADF test while Pedroni uses a variety of tests, including the ADF test. For the Maddala and Wu test, we report the trace test to indicate the number of cointegration relations among variables of a system. Relationships in five models (1-5) (in column 1) were tested region-wise, after incorporating portfolios developed under the high-low correlation strategies. Results are displayed by portfolios, which are based on the correlation between emerging markets *i* from CEE nations and *j* markets from Asia, MENA, Latin America, and the rest of CEE. Blue portfolio captures correlation of 0.2 or lower between these markets; purple portfolio with a correlation between 0.2 and 0.3; green portfolio with a correlation between 0.4 and 0.5; and red portfolio stock having a correlation of 0.6 or more. * denotes rejection of the null hypothesis of no cointegration at 5% or better.

**Table S5-1** VECM Test: Latin America

|  | Intercept | Δ Portfolio ret (-1) | Δ Portfolio ret (-2) | Δ Portfolio ret (-3) | Δ SP500 (-1) | Δ SP500 (-2) | Δ SP500 (-3) | Δ Brent Oil (-1) | Δ Brent Oil (-2) | Δ Brent Oil (-3) | Dummy GFC | Dummy NGFC | ECT (-1) |
| --- | --- | --- | --- | --- | --- | --- | --- | --- | --- | --- | --- | --- | --- |
| ***Model 1:*** ${\Delta P}_{it}=\delta_{2i}+ \theta_{1i}\sum_{k=1}^{n} \Delta P_{jt-k}{+\epsilon}_{it}$ | | | | | | | | | | | | | |
| Yellow portfolio | 5.9411*  (1.8784) | -0.0029  (0.0568) | -0.0167  (0.0579) | - | - | - | - | - | - | - | - | - | -0.0221*  (0.0047) |
| Blue portfolio | 0.0006*  (0.0001) | -0.0061  (0.0063) | -0.0130*  (0.0059) | - | - | - | - | - | - | - | - | - | -0.0002*  (0.0001) |
| Purple portfolio | 0.0078*  (0.0002) | -0.0189  (0.0142) | -0.0046  (0.0140) | - | - | - | - | - | - | - | - | - | -0.0002*  (-2.8353) |
| Green portfolio | 0.0008*  (0.0001) | -0.0029  (0.0040) | -0.0011  (0.0040) | - | - | - | - | - | - | - | - | - | -0.0002*  (0.0001) |
| Red portfolio | 0.0006*  (0.0001) | -0.0005  (0.0025) | -0.0023  (0.0003) | - | - | - | - | - | - | - | - | - | -0.0002*  (0.0001) |
| ***Model 3:*** ${\Delta P}_{it}=\delta_{2i}+ \theta_{1i}\sum_{k=1}^{n} \Delta P_{jt-k}+ \theta_{2i}{\sum_{k=1}^{n} {\Delta BRENT}_{it-k}+}\theta_{3i}\sum_{k=1}^{n} \Delta{S\&P500}_{it-k} {+\delta_{1i}{ECT}_{it-1}+\epsilon}_{it}$, | | | | | | | | | | | | | |
| Yellow portfolio | 7.4263*  (1.9230) | 0.0588  (0.0634) | 0.0139  (0.0644) | 0.0637  (0.0620) | -0.0336  (0.1323) | -0.0840  (0.1386) | -0.1957  (0.1342) | -0.3906  (0.2957) | -0.6019**  (0.3163) | 0.3076  (0.2683) | - | - | -0.0055  (0.0048) |
| Blue portfolio | 0.0005*  (0.0001) | -0.0079  (0.0078) | -0.0111  (0.0082) | 0.0090  (0.0074) | 0.0385*  (0.0110) | -0.0238*  (0.0113) | 0.0305*  (0.0110) | 0.0110*  (0.0054) | -0.0020  (0.0054) | 0.0068  (0.0054) | - | - | 0.0001*  (0.0000) |
| Purple portfolio | 0.0007*  (0.0002) | -0.0074  (0.0163) | -0.0091  (0.0162) | 0.0392*  (0.0158) | 0.0364*  (0.0175) | -0.0272  (0.0176) | 0.0270  (0.0172) | 0.0102  (0.0087) | -0.0017  (0.0086) | 0.0061  (0.0087) | - | - | -0.0001*  (0.0000) |
| Green portfolio | 0.0007*  (0.0001) | -0.0031  (0.0053) | 0.0013  (0.0050) | 0.0084*  (0.0049) | 0.0537*  (0.0097) | -0.0461*  (0.0097) | 0.0391*  (0.0095) | 0.0070  (0.0048) | 0.0047  (0.0048) | 0.0600*  (0.0048) | - | - | -0.0001*  (0.0000) |
| Red portfolio | 0.0007*  (0.0001) | 0.0005  (0.0029) | -0.0021  (0.0029) | 0.0048*  (0.0027) | 0.0185*  (0.0035) | 0.0244*  (0.0035) | 0.0125*  (0.0035) | -0.0001  (0.0001) | 0.0001  (0.0001) | 0.0002*  (0.0001) | - | - | -0.0001*  (0.0000) |
| ***Model 5:*** $P_{it}={\Delta P}_{it}=\delta_{2i}+ \theta_{1i}\sum_{k=1}^{n} \Delta P_{jt-k}+ \theta_{2i}{\sum_{k=1}^{n} \Delta{GFCS\&P500}_{it-k}+\theta}_{3i}\sum_{k=1}^{n} \Delta{NGFCS\&P500}_{it-k} {+\delta_{1i}{ECT}_{it-1}+\epsilon}_{it}$ | | | | | | | | | | | | | |
| Yellow portfolio | 7.3924*  (3.8422) | 0.0578  (0.0633) | 0.0134  (0.0344) | 0.0651  (0.0620) | -0.0401  (0.1324) | -0.0826  (0.1387) | -0.1925  (0.1343) | -0.3951  (0.2957) | -0.6004**  (0.3163) | 0.3095  (0.2684) | -0.1738  (0.1614) | -0.0798  (0.1293) | -0.0055  (0.0048) |
| Blue portfolio | 0.0006*  (0.0001) | -0.0046  (0.0063) | -0.0121*  (0.0060) | 0.0096  (0.0060) | - | - | - | - | - | - | -0.0001  (0.0000) | 0.0001  (0.0023) | -0.0002*  (0.0001) |
| Purple portfolio | 0.0008*  (0.0001) | -0.0147  (0.0140) | -0.0061  (0.0139) | 0.0365*  (0.0137) | - | - | - | - | - | - | 0.0180  (0.0153) | 0.0181  (0.0153) | -0.0002*  (0.0002) |
| Green portfolio | 0.0007*  (0.0001) | -0.0026  (0.0043) | 0.0004  (0.0041) | 0.0080*  (0.0040) | - | - | - | - | - | - | 0.0264*  (0.0086) | 0.0264*  (0.0085) | -0.0003*  (0.0001) |
| Red portfolio | 0.0016*  (0.0001) | -0.0003  (0.0025) | -0.0020  (0.0025) | 0.0056*  (0.0025) | - | - | - | - | - | - | 0.0144*  (0.0033) | 0.0149*  (0.0032) | 0.0149*  (0.0001) |

Note: The table presents three valid VECM models (1), (3) and (5).The dependent variable, ${\Delta P}_{it},$ is each of the six Latin American returns; ${\Delta P}_{jt}$ is a *S1* related portfolio of returns of other countries. The *S1* related portfolios are based on the correlation between emerging markets *i* from LA nations and *j* markets from Asia, MENA, CEE, and the rest of Latin America. Blue portfolio captures correlation of 0.2 or lower; purple portfolio with a correlation between 0.2 and 0.3; green portfolio with a correlation between 0.4 and 0.5; and red portfolio stock having a correlation of 0.6 or more. ${\Delta S\&P500}_{i}$ is the US market price index.$\Delta Brent$ is Brent oil price series and ${\Delta senti}_{i}$ is global investor sentiment. ${S\&P500}_{i}$ effects are examined during the GFC period (*GFC*) and the non-GFC period (*NGFC*) in model (5). These variables appear in first differenced form, represented by $\Delta$. $\delta$ and $\theta s$ are the parameters to be estimated. The error correction term (*ECT*) which is one lag of the residual from equation (1) if significant and negative, confirms a stable long-term relationship between the variables identified. The lag structure for the model is chosen by minimizing the Schwarz Information Criteria. Values in parenthesis are standard errors. * denotes level of significance at 5 percent or better.

**Table S5-2** VECM Test: Asia

|  | Intercept | Δ Portfolio ret (-1) | Δ Portfolio ret (-2) | Δ Portfolio ret (-3) | Δ SP500 (-1) | Δ SP500 (-2) | Δ SP500 (-3) | Δ Brent Oil (-1) | Δ Brent Oil (-2) | Δ Brent Oil (-3) | Δ Sentiment (-1) | Δ Sentiment (-2) | Dummy GFC | Dummy NGFC | ECT (-1) |
| --- | --- | --- | --- | --- | --- | --- | --- | --- | --- | --- | --- | --- | --- | --- | --- |
| ***Model 1:*** ${\Delta P}_{it}=\delta_{2i}+ \theta_{1i}\sum_{k=1}^{n} \Delta P_{jt-k}{+\epsilon}_{it}$ | | | | | | | | | | | | | | | |
| Yellow portfolio | 2.4121*  (0.3285) | 0.0016  (0.0019) | 0.0019  (0.0019) | -0.0010  (0.0019) | - | - | - | - | - | - | - | - | - | - | 0.0770*  (0.0034) |
| Blue portfolio | 0.6933*  (0.1729) | 0.0004  (0.0008) | 0.0002  (0.0008) | -0.0008  (0.0008) | - | - | - | - | - | - | - | - | - | - | 1.2620*  (0.1822) |
| Purple portfolio | 4.1629*  (0.2917) | -0.0023  (0.0021) | 0.0004  (0.0020) | 0.0005  (0.0020) | - | - | - | - | - | - | - | - | - | - | 3.8553*  (0.2421) |
| Green portfolio | 2.9641*  (0.1726) | 0.0018*  (0.0007) | 0.0006  (0.0007) | -0.0010  (0.0007) | - | - | - | - | - | - | - | - | - | - | 2.7458  (0.1417) |
| Red portfolio | 2.2374*  (0.1290) | 0.0163*  (0.0004) | 0.0050*  (0.0005) | 0.0020*  (0.0005) | - | - | - | - | - | - | - | - | - | - | 2.0966*  (0.0961) |
| ***Model 3:*** ${\Delta P}_{it}=\delta_{2i}+ \theta_{1i}\sum_{k=1}^{n} \Delta P_{jt-k}+ \theta_{2i}{\sum_{k=1}^{n} {\Delta BRENT}_{it-k}}\theta_{3i}\sum_{k=1}^{n} \Delta{S\&P500}_{it-k} {+\delta_{1i}{ECT}_{it-1}+\epsilon}_{it}$, | | | | | | | | | | | | | | | |
| Yellow portfolio | 1.9367*  (0.3361) | -0.0002  (0.0019) | 0.0021  (0.0019) | -0.0010  (0.0019) | 0.7585*  (0.0221) | 0.0831*  (0.0223) | 0.0234  (0.0220) | -0.0547  (0.0509) | -0.0071  (0.0539) | - | - | - | - | - | 0.0541*  (0.0036) |
| Blue portfolio | 0.3417  (0.1751) | -0.0039*  (0.0009) | 0.0001  (0.0008) | -0.0008  (0.0009) | 0.6708*  (0.0118) | 0.0488*  (0.0117) | 0.0366*  (0.0116) | -0.0421*  (0.0246) | -0.0160  (0.0247) | - | - | - | - | - | 0.8510*  (0.1929) |
| Purple portfolio | 3.6916*  (0.2968) | -0.0101*  (0.0021) | -0.0001  (0.0021) | 0.0004  (0.0021) | 0.7301*  (0.0200) | 0.2000*  (0.0199) | 0.0494*  (0.0199) | -0.0863*  (0.0401) | -0.0183  (0.0398) | - | - | - | - | - | 3.5056*  (0.2613) |
| Green portfolio | 2.8158*  (0.1748) | -0.0007  (0.0007) | -0.0005  (0.0007) | -0.0015*  (0.0008) | 0.4361*  (0.0118) | 0.2276*  (0.0117) | 0.0255*  (0.0117) | -0.0338  (0.0237) | -0.0174  (0.0236) | - | - | - | - | - | 2.8418*  (0.1517) |
| Red portfolio | 1.7905*  (0.1302) | 0.0075*  (0.0005) | 0.0021*  (0.0005) | 0.0007  (0.0005) | 0.8525*  (0.0088) | 0.5455*  (0.0088) | 0.1385*  (0.0087) | -0.0573*  (0.0180) | 0.0819*  (0.0180) | - | - | - | - | - | 1.5641*  (0.1033) |
| ***Model 4:*** ${\Delta P}_{it}=\delta_{1i}+\theta_{1i}\sum_{k=1}^{n} \Delta P_{jt-k}+ \theta_{2i}\sum_{k=1}^{n} \Delta{S\&P500}_{it-k}{+ \theta_{3i}\sum_{k=1}^{n} \Delta Sentiments}_{it-k}+\mu_{it}$ | | | | | | | | | | | | | | | |
| Yellow portfolio | 2.1960*  (0.3263) | -0.0002  (0.0019) | 0.0022  (0.0019) | -0.0010  (0.0019) | 0.7646*  (0.0216) | 0.1019*  (0.0218) | 0.0117  (0.0217) | - | - | - | 1.7270  (1.4799) | -0.7917  (1.4824) | - | - | 0.0607*  (0.0034) |
| Blue portfolio | 0.5766*  (0.1709) | -0.0039*  (0.0003) | 0.0002  (0.0008) | -0.0008  (0.0008) | 0.6649*  (0.0115) | 0.0551*  (0.0116) | 0.0350*  (0.0116) | - | - | - | 0.169*  (0.0823) | -0.113  (0.0823) | - | - | 1.2379*  (0.1882) |
| Purple portfolio | 3.9530*  (0.2901) | -0.0097*  (0.0020) | -0.0006  (0.0020) | 0.0005  (0.0020) | 0.7272*  (0.0196) | 0.2158*  (0.0198) | 0.0539*  (0.0197) | - | - | - | -0.0035  (0.1306) | 0.0422  (0.1306) | - | - | 3.7473*  (0.2550) |
| Green portfolio | 2.8246*  (0.1720) | -0.0003  (0.0007) | 0.0001  (0.0007) | -0.0010  (0.0007) | 0.4356*  (0.0116) | 0.2347*  (0.0117) | 0.0312*  (0.0117) | - | - | - | -0.0098  (0.0745) | 0.0538  (0.0748) | - | - | 2.8397*  (0.1490) |
| Red portfolio | 2.1086*  (0.1278) | 0.0072*  (0.0004) | 0.0016*  (0.0005) | -0.0004  (0.0005) | 0.8082*  (0.0085) | 0.5203*  (0.0085) | 0.1378*  (0.0085) | - | - | - | 0.2107*  (0.0558) | -0.1518*  (0.0591) | - | - | 1.9090*  (0.1010) |
| ***Model 5:*** $P_{it}={\Delta P}_{it}=\delta_{2i}+ \theta_{1i}\sum_{k=1}^{n} \Delta P_{jt-k}+ \theta_{2i}{\sum_{k=1}^{n} \Delta{GFCS\&P500}_{it-k}+\theta}_{3i}\sum_{k=1}^{n} \Delta{NGFCS\&P500}_{it-k} {+\delta_{1i}{ECT}_{it-1}+\epsilon}_{it}$ | | | | | | | | | | | | | | | |
| Yellow portfolio | 2.2276*  (0.3262) | 0.0019  (0.0019) | 0.0020  (0.0018) | -0.0008  (0.0019) | - | - | - | - | - | - | - | - | 0.7746*  (0.0250) | 0.7817*  (0.0217) | 0.0645*  (0.0034) |
| Blue portfolio | 0.5178*  (0.1686) | 0.0010  (0.0008) | 0.0005  (0.0008) | -0.0005  )0.0008) | - | - | - | - | - | - | - | - | 0.9695*  (0.0130) | 0.9670*  (0.0114) | 1.2633*  (0.1778) |
| Purple portfolio | 4.0213*  (0.2906) | -0.0018  (0.0020) | 0.0008  (0.0020) | 0.0012  )0.0001) | - | - | - | - | - | - | - | - | 0.6224*  (0.0228) | 0.6242*  (0.0197) | 3.7916*  (0.2411) |
| Green portfolio | 2.9316*  (0.1726) | 0.0018*  (0.0007) | 0.0006  (0.0007) | 0.0032  )0.0011) | - | - | - | - | - | - | - | - | 0.0490*  (0.0010) | 0.0926*  (0.0081) | 2.7355*  (0.1417) |
| Red portfolio | 2.2048*  (0.1289) | 0.0111*  (0.0004) | 0.0050*  (0.0005) | 0.0236  )0.0003) | - | - | - | - | - | - | - | - | 0.1556*  (0.0080) | 0.2346*  (0.0059) | 2.0712*  (0.0959) |

Note: The table presents four valid VECM models. Here${\Delta P}_{it}$ is each of the ten Asian countries returns; ${\Delta P}_{jt}$ is a portfolio of returns of other sampled countries, The *S1* related portfolios are based on the correlation between emerging markets *i* from Asia nations and *j* markets from MENA, Latin America, CEE, and the rest of Asia. Blue portfolio captures correlation of 0.2 or lower; purple portfolio with a correlation between 0.2 and 0.3; green portfolio with a correlation between 0.4 and 0.5; and red portfolio stock having a correlation of 0.6 or more. ${\Delta S\&P500}_{i}$ is the US market price index.$\Delta Brent$ is Brent oil price series and ${\Delta senti}_{i}$ is global investor sentiment. ${S\&P500}_{i}$ effects are examined during the GFC period (*GFC*) and the non-GFC period (*NGFC*) in model (5). These variables appear in first differenced form, represented by $\Delta$. $\delta$ and $\theta s$ are the parameters to be estimated. The error correction term (*ECT*) which is one lag of the residual from equation (1) if significant and negative, confirms a stable long-term relationship between the variables identified. The lag structure for the model is chosen by minimizing the Schwarz Information Criteria. Values in parenthesis are standard errors. * denotes level of significance at 5 percent or better.

**Table S5-3** VECM Test: MENA

|  | Intercept | Δ Portfolio ret (-1) | Δ Portfolio ret (-2) | Δ Portfolio ret (-3) | Δ SP500 (-1) | Δ SP500 (-2) | Δ SP500 (-3) | Δ Brent Oil (-1) | Δ Brent Oil (-2) | Δ Brent Oil (-3) | Δ Sentiment (-1) | Δ Sentiment (-2) | Dummy GFC | Dummy NGFC | ECT (-1) |
| --- | --- | --- | --- | --- | --- | --- | --- | --- | --- | --- | --- | --- | --- | --- | --- |
| ***Model 1:*** ${\Delta P}_{it}=\delta_{2i}+ \theta_{1i}\sum_{k=1}^{n} \Delta P_{jt-k}{+\epsilon}_{it}$ | | | | | | | | | | | | | | | |
| Yellow portfolio | -0.6394*  (0.1169) | 0.0023*  (0.0005) | 0.0007  (0.0005) | -0.0004  (0.0005) | - | - | - | - | - | - | - | - | - | - | 0.1146*  (0.0029) |
| Blue portfolio | -0.0001  (0.0000) | 0.0245*  (0.0023) | 0.0066*  (0.0022) | 0.0053  (0.0022) | - | - | - | - | - | - | - | - | - | - | -0.0011*  (0.0003) |
| Purple portfolio | 0.3105*  (0.1370) | 0.0044*  (0.0009) | 0.0029*  (0.0009) | 0.0019*  (0.0009) | - | - | - | - | - | - | - | - | - | - | 0.0077  (0.1125) |
| Green portfolio | 0.4345*  (0.1169) | 0.0040*  (0.0005) | 0.0015*  (0.0005) | 0.0015*  (0.0005) | - | - | - | - | - | - | - | - | - | - | 0.0706  (0.1018) |
| Red portfolio | 0.4000*  (0.0491) | 0.0036*  (0.0002) | 0.0016*  (0.0002) | 0.0008*  (0.0002) | - | - | - | - | - | - | - | - | - | - | -0.0680*  (0.0404) |
| ***Model 2:*** $\Delta P_{it}=\delta_{1i}+ \theta_{1i}\Delta P_{jt-k}+ \theta_{2i}{\Delta S\&P500}_{jt-k}+\mu_{it}$ | | | | | | | | | | | | | | | |
| Yellow portfolio | -0.7155*  (0.1149) | -0.0010*  (0.0005) | 0.0010**  (0.0005) | -0.0004  (0.0005) | 0.5322*  (0.0078) | 0.0130  (0.0080) | 0.0189*  (0.0078) | - | - | - | - | - | - | - | 0.0858*  (0.0030) |
| Blue portfolio | -0.0001*  (0.0000) | 0.0008  (0.0003) | 0.0002  (0.0023) | 0.0028  (0.0023) | 0.0002*  (0.0000) | 0.0001*  (0.0000) | 0.0001*  (0.0000) | - | - | - | - | - | - | - | 0.0001*  (0.0000) |
| Purple portfolio | 0.2367*  (0.1365) | 0.0024*  (0.0009) | 0.0022*  (0.0009) | 0.0016*  (0.0009) | 0.2736*  (0.0092) | 0.1153*  (0.0093) | 0.0800*  (0.0091) | - | - | - | - | - | - | - | -0.0353*  (0.1128) |
| Green portfolio | 0.3635*  (0.0064) | 0.0012*  (0.0005) | 0.0056  (0.0005) | 0.0013*  (0.0004) | 0.3368*  (0.0097) | 0.1016*  (0.0080) | 0.0694*  (0.0079) | - | - | - | - | - | - | - | 0.0151  (0.1020) |
| Red portfolio | 0.3478*  (0.0499) | 0.0018*  (0.0002) | 0.0010*  (0.0002) | 0.0005*  (0.0001) | 0.2334*  (0.0033) | 0.0851*  (0.0033) | 0.0696*  (0.0033) | - | - | - | - | - | - | - | 0.0355*  (0.0410) |
| ***Model 3:*** ${\Delta P}_{it}=\delta_{1i}+ {\Delta\theta}_{1i}P_{jt-k}+ \theta_{2i}\Delta{S\&P500}_{it-k}+\theta_{3i}{\Delta Brent}_{it-k}+\mu_{it}$ | | | | | | | | | | | | | | | |
| Yellow portfolio | -0.8131*  (0.1170) | -0.0012*  (0.0005) | 0.0010**  (0.0005) | -0.0001  (0.0005) | 0.5397*  (0.0079) | 0.0158**  (0.0081) | 0.0178**  (0.0078) | -0.0465*  (0.0164) | -0.0066  (0.0165) | - | - | - | - | - | 0.0915*  (0.0031) |
| Blue portfolio | -0.0001  (0.0000) | -0.0007  (0.0025) | 0.0011  (0.0024) | 0.0038  (0.0024) | 0.0002*  (0.0000) | 0.0001*  (0.0000) | 0.0001*  (0.0000) | 0.0001  (0.0000) | 0.0001  (0.0000) | - | - | - | - | - | -0.0011*  (0.0003) |
| Purple portfolio | 0.3195  (0.1387) | 0.0022  (0.0009) | 0.0019  (0.0009) | 0.0014  (0.0009) | 0.2587*  (0.0093) | 0.1157*  (0.0093) | 0.0723*  (0.0092) | 0.1140*  (0.0195) | 0.0426*  (0.0197) | - | - | - | - | - | 0.0017  (0.0047) |
| Green portfolio | 0.4716*  (0.1176) | 0.0011*  (0.0005) | 0.0005  (0.0004) | 0.0013*  (0.0005) | 0.3280*  (0.0080) | 0.1050*  (0.0080) | 0.0626*  (0.0079) | 0.0696*  (0.0163) | 0.0576*  (0.0164) | - | - | - | - | - | 0.0521  (0.1030) |
| Red portfolio | 0.4733*  (0.0493) | 0.0014*  (0.0002) | 0.0011*  (0.0002) | 0.0004*  (0.0002) | 0.2290*  (0.0334) | 0.0831*  (0.0033) | 0.0633*  (0.0033) | 0.0463*  (0.0069) | 0.0344*  (0.0069) | - | - | - | - | - | 0.1202*  (0.0412) |
| ***Model 4:*** $\Delta P_{it-k}=\delta_{1i}+ \theta_{1i}{\Delta P}_{jt-k}+ \theta_{2i}{\Delta S\&P500}_{it-k}{+ \theta}_{3i}\Delta{sentiments}_{it-k}+\mu_{it}$ | | | | | | | | | | | | | | | |
| Yellow portfolio | -0.7156*  (0.1149) | -0.0010**  (0.0005) | 0.0010**  (0.0005) | -0.0004  (0.0005) | 0.5323*  (0.0078) | 0.0133***  (0.0080) | 0.0189**  (0.0078) | - | - | - | 1.0295**  (0.5170) | -0.9197**  (0.5173) | - | - | 0.0858*  (0.0029) |
| Blue portfolio | -0.0001*  (0.0000) | 0.0008  (0.0024) | 0.0001  (0.0023) | 0.0028  (0.0022) | 0.0002*  (0.0000) | 0.0001*  (0.0000) | 0.0001*  (0.0000) | - | - | - | -0.0202  (0.0185) | 0.0352*  (0.0185) | - | - | -0.0011*  (0.0003) |
| Purple portfolio | 0.2367*  (0.1365) | 0.0024*  (0.0009) | 0.0022*  (0.0009) | 0.0016*  (0.0009) | 0.2735*  (0.0092) | 0.1150*  (0.0092) | 0.0800*  (0.0092) | - | - | - | -0.0669  (0.0598) | 0.0749  (0.0598) | - | - | -0.033*  (0.1128) |
| Green portfolio | 0.3636  (0.1164) | 0.0022  (0.0005) | 0.0006  (0.0005) | 0.0013  (0.0005) | 0.3368  (0.0079) | 0.1013  (0.0080) | 0.0693  (0.0079) | - | - | - | -0.0625  (0.0506) | 0.0666  (0.0506) | - | - | 0.0523  (0.1020) |
| Red portfolio | 0.3481*  (0.0489) | 0.0018*  (0.0002) | 0.0010*  (0.0002) | 0.0005*  (0.0002) | 0.2333*  (0.0033) | 0.0847*  (0.0033) | 0.0697*  (0.0033) | - | - | - | -0.1326*  (0.0212) | 0.1104*  (0.0213) | - | - | 0.0345  (0.0410) |
| ***Model 5:*** ${\Delta P}_{it}=\delta_{1i}+ \theta_{1i}{\Delta P}_{jt-k}+ \theta_{2i}\Delta GFC*{S\&P500}_{it-k}+\theta_{3i}\Delta NGFC*{S\&P500}_{it-k}{+\mu}_{it}$ | | | | | | | | | | | | | | | |
| Yellow portfolio | -0.7124*  (0.1156) | 0.0025*  (0.0005) | 0.0009**  (0.0005) | -0.0003  (0.0005) | - | - | - | - | - | - | - | - | 0.4719*  (0.0088) | 0.4261*  (0.0078) | 0.0870*  (0.0029) |
| Blue portfolio | 0.0001*  (0.0000) | 0.0264*  (0.0023) | 0.0077*  (0.0022) | 0.0055*  (0.0024) | - | - | - | - | - | - | - | - | 0.1697*  (0.0034) | 0.1687*  (0.0034) | -0.0011*  (0.0023) |
| Purple portfolio | 0.3001*  (0.1370) | 0.0043*  (0.0009) | 0.0030*  (0.0008) | 0.0002*  (0.0008) | - | - | - | - | - | - | - | - | 0.1105*  (1.7334) | 0.0254*  (0.0010) | -0.0429*  (0.1132) |
| Green portfolio | 0.4213*  (0.1169) | 0.0040*  (0.0005) | 0.0015*  (0.0005) | 0.0016*  (0.0005) | - | - | - | - | - | - | - | - | 0.1251*  (1.4888) | 0.0638*  (0.0008) | 0.0141  (0.1025) |
| Red portfolio | 0.3928*  (0.0491) | 0.0036*  (0.0002) | 0.0016*  (0.0002) | 0.0008*  (0.0002) | - | - | - | - | - | - | - | - | 0.0855*  (0.6270) | 0.0411*  (0.0034) | 0.0297  (0.0412) |

Note: The table presents all five VECM models. The dependent variable,${\Delta P}_{i}$, is each of the nine MENA countries returns. ${\Delta P}_{j}$ is a *S1* related portfolio of returns of other sampled countries. The *S1* related portfolios are based on the correlation between emerging markets *i* from MENA nations and *j* markets from Asia, Latin America, CEE, and the rest of MENA. Blue portfolio captures correlation of 0.2 or lower; purple portfolio with a correlation between 0.2 and 0.3; green portfolio with a correlation between 0.4 and 0.5; and red portfolio stock having a correlation of 0.6 or more. ${\Delta S\&P500}_{i}$ is the US market price index.$\Delta Brent$ is Brent oil price series and ${\Delta senti}_{i}$ is global investor sentiment. ${S\&P500}_{i}$ effects are examined during the GFC period (*GFC*) and the non-GFC period (*NGFC*) in model (5). These variables appear in first differenced form, represented by $\Delta$. $\delta$ and $\theta s$ are the parameters to be estimated. The error correction term (*ECT*) which is one lag of the residual from equation (1) if significant and negative, confirms a stable long-term relationship between the variables identified. The lag structure for the model is chosen by minimizing the Schwarz Information Criteria. Values in parenthesis are standard errors. * denotes level of significance at 5 percent or better.

**Table S5-4** VECM Test: CEE

|  | Intercept | Δ Portfolio ret (-1) | Δ Portfolio ret (-2) | Δ Portfolio ret (-3) | Δ SP500 (-1) | Δ SP500 (-2) | Δ SP500 (-3) | Δ Brent Oil (-1) | Δ Brent Oil (-2) | Δ Brent Oil (-3) | Δ Sentiment (-1) | Δ Sentiment (-2) | Dummy GFC | Dummy NGFC | ECT (-1) |
| --- | --- | --- | --- | --- | --- | --- | --- | --- | --- | --- | --- | --- | --- | --- | --- |
| ***Model 1:*** $\Delta P_{it}=\delta_{1i}+ \theta_{1i}\Delta P_{jt-k}+\mu_{it}$ | | | | | | | | | | | | | | | |
| Yellow portfolio | 2.9700**  (1.2811) | 0.0023  (0.0045) | 0.0004  (0.0045) | 0.0003  (0.0045) | - | - | - | - | - | - | - | - | - | - | -0.0001  (0.9790) |
| Blue portfolio | 3.1951*  (0.8445) | 0.0126*  (0.0064) | -0.0009  (0.0063) | 0.0007  (0.0063) | - | - | - | - | - | - | - | - | - | - | 1.7813*  (0.4931) |
| Purple portfolio | 2.5415*  (0.9129) | 0.0164*  (0.0039) | 0.0019  (0.0039) | 0.0005  (0.0040) | - | - | - | - | - | - | - | - | - | - | 2.5769*  (0.6095) |
| Green portfolio | 3.2619*  (0.8868) | 0.0087  (0.0058) | 0.0002  (0.0057) | -0.0075  (0.0058) | - | - | - | - | - | - | - | - | - | - | 1.6184*  (0.4979) |
| Red portfolio | 3.8421*  (0.3453) | 0.0006*  (0.0014) | 0.0007  (0.0014) | 0.0007  (0.0014) | - | - | - | - | - | - | - | - | - | - | 1.6200*  (0.1799) |
| ***Model 2:*** ${\Delta P}_{it}=\delta_{1i}+ \theta_{1i}\Delta P_{jt-k}+ \theta_{2i}\Delta{S\&P500}_{it-k}+\mu_{it}$ | | | | | | | | | | | | | | | |
| Yellow portfolio | 2.2673**  (1.2739) | -0.0101**  (0.0045) | -0.0004  (0.0044) | 0.0005  (0.0045) | 2.3148*  (0.0822) | 0.3148*  (0.0832) | 0.1044  (0.0823) | - | - | - | - | - | - | - | -0.0186*  (0.0039) |
| Blue portfolio | 2.6325*  (0.8380) | -0.0010  (0.0064) | -0.0042  (0.0064) | -0.0002  (0.0063) | 2.5921*  (0.0558) | 0.2041*  (0.0560) | 0.1326*  (0.0558) | - | - | - | - | - | - | - | 1.9188*  (0.4942) |
| Purple portfolio | 1.6018*  (0.8988) | -0.0181*  (0.0039) | -0.0147*  (0.0039) | 0.0011  (0.0039) | 2.6387*  (0.0594) | 2.0984*  (0.0598) | 0.2955*  (0.0596) | - | - | - | - | - | - | - | 2.6640*  (0.6005) |
| Green portfolio | 2.7854*  (0.8806) | -0.0020  (0.0058) | -0.0005  (0.0058) | -0.0074  (0.0058) | 2.4640*  (0.0594) | 0.2159*  (0.0595) | 0.1908*  (0.0593) | - | - | - | - | - | - | - | 1.7823*  (0.5032) |
| Red portfolio | 3.3878*  (0.3420) | -0.0118*  (0.0014) | 0.0006  (0.0014) | 0.0006  (0.0013) | 3.0774*  (0.0231) | 0.2067*  (0.0233) | 0.1897*  (0.0232) | - | - | - | - | - | - | - | 1.7349*  (0.1804) |
| ***Model 3:*** ${\Delta P}_{it}=\delta_{1i}+ \theta_{1i}\Delta P_{jt-k}+ \theta_{2i}{\Delta S\&P500}_{it-k}+\theta_{3i}\Delta{Brent}_{it-k}+\mu_{it}$ | | | | | | | | | | | | | | | |
| Yellow portfolio | 2.2903***  (1.3375) | -0.0108**  (0.0147) | 0.0003  (0.9572) | 0.0008  (0.0047) | 2.3326*  (0.0855) | 0.2791*  (0.0867) | 0.0988  (0.0850) | 0.1265  (0.2093) | 0.7026*  (0.2303) | 0.3924**  (0.2075) | - | - | - | - | -0.0213*  (0.0041) |
| Blue portfolio | 2.6219*  (0.8379) | -0.0091  (0.0064) | -0.0003  (0.0064) | -0.0003  (0.0064) | 2.5946*  (0.0558) | 0.1970*  (0.0560) | 0.1320*  (0.0558) | -0.1180*  (0.0420) | -0.1575*  (0.0461) | -0.2827*  (0.0417) | - | - | - | - | 2.0102*  (0.4969) |
| Purple portfolio | 1.5987*  (0.8988) | -0.0182*  (0.0039) | -0.0146*  (0.0039) | 0.0011  (0.0039) | 2.6371*  (0.0594) | 2.1024*  (0.0598) | 0.2980*  (0.0596) | 0.1528*  (0.0431) | 0.0413  (0.0474) | -0.0035  (0.0431) | - | - | - | - | 2.6939*  (0.6004) |
| Green portfolio | 2.7881*  (0.8805) | -0.0018  (0.0058) | -0.0005  (0.0058) | -0.0075  (0.0058) | 2.4656*  (0.0594) | 0.2093*  (0.0596) | 0.1902*  (0.0594) | -0.0993*  (0.0450) | -0.1491  (0.0495) | -0.2507*  (0.0448) | - | - | - | - | 1.8857*  (0.5064) |
| Red portfolio | 3.3833*  (0.3419) | -0.0117*  (0.0014) | 0.0007  (0.0014) | 0.0006  (0.0014) | 3.0801*  (0.0231) | 0.1981*  (0.0232) | 0.1895*  (0.0232) | -0.1296*  (0.0177) | -0.1641*  (0.0195) | -0.3340*  (0.0177) | - | - | - | - | 1.8153*  (0.1812) |
| ***Model 4:*** ${\Delta P}_{it}=\delta_{1i}+ \theta_{1i}{\Delta P}_{jt-k}+ \theta_{2i}{\Delta S\&P500}_{it-k}{+ \theta}_{3i}{\Delta sentiments}_{it-k}+\mu_{it}$ | | | | | | | | | | | | | | | |
| Yellow portfolio | 2.2739**  1.2739 | -0.0102**  0.0045 | -0.0004  0.0045 | 0.0005  0.0045 | 2.3128*  0.0822 | 0.3113*  0.0832 | 0.1047  0.0823 | - | - | - | -5.0247  5.2463 | 7.4306  5.2485 | - | - | -0.0186*  0.0040 |
| Blue portfolio | 2.6358*  (0.8380) | -0.0093  (0.0064) | -0.0004  (0.0064) | -0.0002  (0.0063) | 2.5904*  (0.0558) | 0.2031*  (0.0561) | 0.1331*  (0.0559) | - | - | - | 0.0908  (0.0372) | 0.0280  (0.0372) | - | - | 1.9229*  (0.4942) |
| Purple portfolio | 1.6032*  (0.8988) | -0.0182*  (0.0039) | -0.0147*  (0.0039) | 0.0012  (0.0039) | 2.6376*  (0.0594) | 2.0940*  (0.0598) | 0.2955*  (0.0596) | - | - | - | -0.0119*  (0.0374) | 0.0102*  (0.0374) | - | - | 2.6634*  (0.6000) |
| Green portfolio | 2.7813*  (0.8806) | -0.0020  (0.0058) | -0.0005  (0.0058) | -0.0074  (0.0058) | 2.4622*  (0.0594) | 0.2141*  (0.0600) | 0.1913*  (0.0593) | - | - | - | 0.0182  (0.0381) | 0.0369  (0.0382) | - | - | 1.7806*  (0.5032) |
| Red portfolio | 3.3845*  (0.3419) | -0.0119*  (0.0014) | 0.0006  (0.0014) | 0.0005  (0.0014) | 0.3075*  (0.0231) | 0.0240*  (0.0233) | 0.1902*  (0.0232) | - | - | - | -0.0245*  (0.1493) | 0.0686*  (0.1495) | - | - | 1.7370*  (0.1804) |
| ***Model 5:*** $\Delta P_{it}=\delta_{1i}+ \theta_{1i}\Delta P_{jt-k}+ \theta_{2i}\Delta GFC*{S\&P500}_{it}+\theta_{3i}\Delta NGFC*{S\&P500}_{it}{+\mu}_{it}$ | | | | | | | | | | | | | | | |
| Yellow portfolio | 2.0918***  (1.2665) | 0.0036  (0.0044) | 0.0015  (0.0044) | 0.0010  (0.0044) | - | - | - | - | - | - | - | - | 3.1650*  (0.0901) | 3.1920*  (0.0817) | -0.0159*  (0.0039) |
| Blue portfolio | 2.8474*  (0.8383) | 0.0135*  (0.0064) | 0.0012  (0.0063) | 0.0010  (0.0063) | - | - | - | - | - | - | - | - | 0.0297*  (0.8629) | 0.2027*  (0.0453) | 1.8421*  (0.4945) |
| Purple portfolio | 2.5383*  (0.9130) | 0.0163*  (0.0093) | 0.0020*  (0.0039) | 0.0005*  (0.0039) | - | - | - | - | - | - | - | - | -0.0158  (0.1119) | -0.0077  (0.0615) | 2.5762*  (0.6096) |
| Green portfolio | 2.5650*  (0.8749) | 0.0097*  (0.0057) | 0.0027  (0.0057) | -0.0041  (0.0057) | - | - | - | - | - | - | - | - | 3.4026*  (0.0673) | 3.3892*  (0.0592) | 1.6261*  (0.4999) |
| Red portfolio | 3.0654*  (0.3404) | 0.0082*  (0.0014) | 0.0014  (0.0013) | 0.0024  (0.0014) | - | - | - | - | - | - | - | - | 0.5859*  (0.0434) | 0.0381*  (0.0236) | 1.5831*  (0.1796) |

Note: The table presents all five VECM models. The dependent variable,${\Delta P}_{it},$ is each of the twelve CEE countries returns; ${\Delta P}_{jt}$ is a S1 related portfolio of returns of other sampled countries. The *S1* related portfolios are based on the correlation between emerging markets *i* from CEE nations and *j* markets from Asia, Latin America, MENA, and the rest of CEE. Blue portfolio captures correlation of 0.2 or lower; purple portfolio with a correlation between 0.2 and 0.3; green portfolio with a correlation between 0.4 and 0.5; and red portfolio stock having a correlation of 0.6 or more. ${\Delta S\&P500}_{i}$ is the US market price index.$\Delta Brent$ is Brent oil price series and ${\Delta senti}_{i}$ is global investor sentiment. ${S\&P500}_{i}$ effects are examined during the GFC period (*GFC*) and the non-GFC period (*NGFC*) in model (5). These variables appear in first differenced form, represented by $\Delta$. $\delta$ and $\theta s$ are the parameters to be estimated. The error correction term (*ECT*) which is one lag of the residual from equation (1) if significant and negative, confirms a stable long-term relationship between the variables identified. The lag structure for the model is chosen by minimizing the Schwarz Information Criteria. Values in parenthesis are standard errors. * denotes level of significance at 5 percent or better.

**Table S6-1** Long-term regression results: Latin America

|  | $P_{jt}$ | S&P500 | Brent | Sentiment | GFC | NGFC |
| --- | --- | --- | --- | --- | --- | --- |
| ***Model 1:*** $P_{it}=\delta_{1i}+ \theta_{1i}P_{jt}+\mu_{it}$ | | | | | | |
| Blue portfolio | 0.0538*  (0.0157) | - | - | - | - | - |
| Purple portfolio | 1.0576*  (0.0182) | - | - | - | - | - |
| Green portfolio | 0.9041*  (0.0065) | - | - | - | - | - |
| Red portfolio | 0.8863*  (0.0027) | - | - | - | - | - |
| ***Model 2:*** $P_{it}=\delta_{1i}+ \theta_{1i}P_{jt}+ \theta_{2i}{S\&P500}_{it}+\mu_{it}$ | | | | | | |
| Blue portfolio | 0.1147*  (0.0134) | 0.0012*  (0.0000) | - | - | - | - |
| Purple portfolio | 0.8662*  (0.0167) | 0.0009*  (0.0001) | - | - | - | - |
| Green portfolio | 0.7521*  (0.0070) | 0.0006*  (0.0001) | - | - | - | - |
| Red portfolio | 0.8993*  (0.0033) | -0.0001*  (0.0000) | - | - | - | - |
| ***Model 3:*** $P_{it}=\delta_{1i}+ \theta_{1i}P_{jt}+ \theta_{2i}{S\&P500}_{it}+\theta_{3i}{Brent}_{it}+\mu_{it}$ | | | | | | |
| Blue portfolio | -0.0128*  (0.0117) | 0.0011*  (0.0001) | 0.0126*  (0.0002) | - | - | - |
| Purple portfolio | 0.6562  (0.0209) | 0.0009*  (0.0001) | 0.0058*  (0.0004) | - | - | - |
| Green portfolio | 0.5611*  (0.0078) | 0.0007*  (0.0001) | 0.0077*  (0.0002) | - | - | - |
| Red portfolio | 0.7874*  (0.0043) | 0.0001*  (0.0000) | 0.0034*  (0.0001) | - | - | - |
| ***Model 4:*** $P_{it}=\delta_{1i}+ \theta_{1i}P_{jt}+ \theta_{2i}{S\&P500}_{it}{+ \theta}_{3i}{sentiments}_{it}+\mu_{it}$ | | | | | | |
| Blue portfolio | 0.1325*  (0.0134) | 0.0012*  (0.0000) | - | 0.7537*  (0.0591) | - | - |
| Purple portfolio | 0.8827*  (0.0165) | 0.0008*  (0.0001) | - | 0.9189*  (0.0695) | - | - |
| Green portfolio | 0.7497*  (0.0070) | 0.0006*  (0.0001) | - | 0.5544*  (0.0377) | - | - |
| Red portfolio | 0.8949*  (0.0033) | -0.0001*  (0.0000) | - | 0.3233*  (0.0164) | - | - |
| ***Model 5:*** $P_{it}=\delta_{1i}+ \theta_{1i}P_{jt}+ \theta_{2i}GFC*{S\&P500}_{it}+\theta_{3i}NGFC*{S\&P500}_{it}{+\mu}_{it}$ | | | | | | |
| Blue portfolio | 0.0461*  (0.0135) | - | - | - | 1.8958*  (0.0286) | 1.8165*  (0.0272) |
| Purple portfolio | 0.8569*  0.0180 | - | - | - | 1.1763*  (0.0382) | 1.1638*  (0.0360) |
| Green portfolio | 0.7469*  (0.0072) | - | - | - | 0.9013*  (0.0213) | 0.8681*  (0.0202) |
| Red portfolio | 0.9026*  (0.0034) | - | - | - | -0.0609*  (0.0099) | -0.0822*  (0.0095) |

Note: This table displays the long-term relationships depicted in models (1-5). The dependent variable, $P_{it},$ is a panel of Latin American stock markets. $P_{jt}$ is a *S1* related portfolio of other sampled stock markets. Blue portfolio captures correlation of 0.2 or lower; purple portfolio with a correlation between 0.2 and 0.3; green portfolio with a correlation between 0.4 and 0.5; and red portfolio stock having a correlation of 0.6 or more. ${S\&P500}_{it}$ is the US market price index; ${Brent}_{it}$ is Brent oil price series; and ${senti}_{it}$ is global investor sentiment.${S\&P500}_{i}$ effects are examined during the GFC period (*GFC*) and the non-GFC period (*NGFC*) in model 5. * denotes significance at 5 percent or better.

**Table S6-2** Long-term regression results: Asia

|  | $P_{jt}$ | SP500 | Brent Oil | Sentiment | Dummy GFC | Dummy NGFC |
| --- | --- | --- | --- | --- | --- | --- |
| ***Model 1:*** $P_{it}=\delta_{1i}+ \theta_{1i}P_{jt}+\mu_{it}$ | | | | | | |
| Blue portfolio | 0.0336*  (0.0060) | - | - | - | - | - |
| Purple portfolio | 0.6480*  (0.0092) | - | - | - | - | - |
| Green portfolio | 0.6778*  (0.0054) | - | - | - | - | - |
| Red portfolio | 0.8654*  (0.0022) | - | - | - | - | - |
| ***Model 2:*** $P_{it}=\delta_{1i}+ \theta_{1i}P_{jt}+ \theta_{2i}{S\&P500}_{it}+\mu_{it}$ | | | | | | |
| Blue portfolio | -0.1817*  (0.0054) | 1.1878*  (0.0129) | - | - | - | - |
| Purple portfolio | 0.4562*  (0.0083) | 1.2081*  (0.0164) | - | - | - | - |
| Green portfolio | 0.4764*  (0.0054) | 1.0033*  (0.0120) | - | - | - | - |
| Red portfolio | 0.7026*  (0.0024) | 0.7766*  (0.0066) | - | - | - | - |
| ***Model 3:*** $P_{it}=\delta_{1i}+ \theta_{1i}P_{jt}+ \theta_{2i}{S\&P500}_{it}+\theta_{3i}{Brent}_{it}+\mu_{it}$ | | | | | | |
| Blue portfolio | -0.2277*  (0.0053) | 1.2074*  (0.0125) | 0.1126*  (0.0033) | - | - | - |
| Purple portfolio | 0.4143*  (0.0088) | 1.2269*  (0.0163) | 0.0828*  (0.0060) | - | - | - |
| Green portfolio | 0.4383*  (0.0057) | 1.0317*  (0.0120) | 0.0857*  (0.0041) | - | - | - |
| Red portfolio | 0.6727*  (0.0026) | 0.8104*  (0.0066) | 0.0671*  (0.0021) | - | - | - |
| ***Model 4:*** $P_{it}=\delta_{1i}+ \theta_{1i}P_{jt}+ \theta_{2i}{S\&P500}_{it}{+ \theta}_{3i}{sentiments}_{it}+\mu_{it}$ | | | | | | |
| Blue portfolio | -0.1822*  (0.0053) | 1.1868*  (0.0129) | - | 0.0801*  (0.0245) | - | - |
| Purple portfolio | 0.4575*  (0.0083) | 1.2061*  (0.0164) | - | 0.1378*  (0.0337) | - | - |
| Green portfolio | 0.4764*  (0.0054) | 1.0033*  (0.0120) | - | 0.0001  (0.0230) | - | - |
| Red portfolio | 0.7005*  (0.0024) | 0.7776*  (0.0066) | - | 0.1210*  (0.0116) | - | - |
| ***Model 5:*** $P_{it}=\delta_{1i}+ \theta_{1i}P_{jt}+ \theta_{2i}GFC*{S\&P500}_{it}+\theta_{3i}NGFC*{S\&P500}_{it}{+\mu}_{it}$ | | | | | | |
| Blue portfolio | -0.1941*  (0.0053) | - | - | - | 1.2431*  (0.0139) | 1.2210*  (0.0133) |
| Purple portfolio | 0.4439*  (0.0085) | - | - | - | 1.2606*  (0.0182) | 1.2442*  (0.0172) |
| Green portfolio | 0.4666*  (0.0055) | - | - | - | 1.0550*  (0.0133) | 1.0396*  (0.0127) |
| Red portfolio | 0.7049*  (0.0025) | - | - | - | 0.7724*  (0.0071) | 0.7645*  (0.0067) |

Note: This table displays the long-term relationships depicted models (1-5). The dependent variable, $P_{it},$ is a panel of Asian stock markets. $P_{jt}$ is a S1 related portfolio of other sampled stock markets.$P_{jt}$ is a *S1* related portfolio of other sampled stock markets. Blue portfolio captures correlation of 0.2 or lower; purple portfolio with a correlation between 0.2 and 0.3; green portfolio with a correlation between 0.4 and 0.5; and red portfolio stock having a correlation of 0.6 or more. ${S\&P500}_{it}$ is the US market price index; ${Brent}_{it}$ is Brent oil price series; and ${senti}_{it}$ is global investor sentiment.${S\&P500}_{i}$ effects are examined during the GFC period (*GFC*) and the non-GFC period (*NGFC*) in model 5. * denotes significance at 5 percent or better.

**Table S6-3** Long-term regression results: MENA

|  | $P_{jt}$ | SP500 | Brent Oil | Sentiment | Dummy GFC | Dummy NGFC |
| --- | --- | --- | --- | --- | --- | --- |
| ***Model 1:*** $P_{it}=\delta_{1i}+ \theta_{1i}P_{jt}+\mu_{it}$ | | | | | | |
| Blue portfolio | 0.0204  (0.0075) | - | - | - | - | - |
| Purple portfolio | 0.3633*  (0.0047) | - | - | - | - | - |
| Green portfolio | 0.5091*  (0.0036) | - | - | - | - | - |
| Red portfolio | 0.7485*  (0.0022) | - | - | - | - | - |
| ***Model 2:*** $P_{it}=\delta_{1i}+ \theta_{1i}P_{jt}+ \theta_{2i}{S\&P500}_{it}+\mu_{it}$ | | | | | | |
| Blue portfolio | 0.0192*  (0.0080) | 0.0019*  (0.0031) | - | - | - | - |
| Purple portfolio | 0.3687*  (0.0053) | -0.0302*  (0.0145) | - | - | - | - |
| Green portfolio | 0.5676*  (0.0044) | -0.2773*  (0.0120) | - | - | - | - |
| Red portfolio | 0.7102*  (0.0025) | 0.1980*  (0.0062) | - | - | - | - |
| ***Model 3:*** $P_{it}=\delta_{1i}+ \theta_{1i}P_{jt}+ \theta_{2i}{S\&P500}_{it}+\theta_{3i}{Brent}_{it}+\mu_{it}$ | | | | | | |
| Blue portfolio | -0.0252*  (0.0080) | 0.0031  (0.0030) | 0.0275*  (0.0011) | - | - | - |
| Purple portfolio | 0.3228*  (0.0045) | 0.0548*  (0.0019) | 0.1503*  (0.0035) | - | - | - |
| Green portfolio | 0.5234*  (0.0045*) | -0.2264*  (0.0118) | 0.1091*  (0.0037) | - | - | - |
| Red portfolio | 0.6546*  (0.0026) | 0.2437*  (0.0062) | 0.1148*  (0.0021) | - | - | - |
| ***Model 4:*** $P_{it}=\delta_{1i}+ \theta_{1i}P_{jt}+ \theta_{2i}{S\&P500}_{it}{+ \theta}_{3i}{sentiments}_{it}+\mu_{it}$ | | | | | | |
| Blue portfolio | 0.0193*  (0.0080) | 0.0020  (0.0031) | - | -0.0076*  (0.0063) | - | - |
| Purple portfolio | 0.3590*  (0.0045) | 0.0648*  (0.0020) | - | 0.0965*  (0.0263) | - | - |
| Green portfolio | 0.5603*  (0.0043) | 0.0305*  (0.0023) | - | -0.0002*  (0.0000) | - | - |
| Red portfolio | 0.7107*  (0.0025) | 0.1980*  (0.0062) | - | 0.0022  (0.0116) | - | - |
| ***Model 5:*** $P_{it}=\delta_{1i}+ \theta_{1i}P_{jt}+ \theta_{2i}GFC*{S\&P500}_{it}+\theta_{3i}NGFC*{S\&P500}_{it}{+\mu}_{it}$ | | | | | | |
| Blue portfolio | 0.0009  (0.0080) | - | - | - | 0.0259*  (0.0034) | 0.0171*  (0.0032) |
| Purple portfolio | 0.3584*  (0.0052) | - | - | - | 0.0640*  (0.0029) | 0.0001  (0.0000) |
| Green portfolio | 05630  (0.0043) | - | - | - | 0.0305*  (0.0023) | -0.0002*  (0.0001) |
| Red portfolio | 0.6903*  (0.0025) | - | - | - | 0.0797*  (0.0013) | 0.0002*  (0.0001) |

Note: This table displays the long-term relationships depicted models (1-5). The dependent variable, $P_{it},$ is a panel of MENA stock markets. $P_{jt}$ is a S1 related portfolio of other sampled stock markets.$P_{jt}$ is a *S1* related portfolio of other sampled stock markets. Blue portfolio captures correlation of 0.2 or lower; purple portfolio with a correlation between 0.2 and 0.3; green portfolio with a correlation between 0.4 and 0.5; and red portfolio stock having a correlation of 0.6 or more. ${S\&P500}_{it}$ is the US market price index; ${Brent}_{it}$ is Brent oil price series; and ${senti}_{it}$ is global investor sentiment.${S\&P500}_{i}$ effects are examined during the GFC period (*GFC*) and the non-GFC period (*NGFC*) in model 5. * denotes significance at 5 percent or better.

**Table S6-4** Long-term regression results: CEE

|  | $P_{jt}$ | SP500 | Brent Oil | Sentiment | Dummy GFC | Dummy NGFC |
| --- | --- | --- | --- | --- | --- | --- |
| ***Model 1:*** $P_{it}=\delta_{1i}+ \theta_{1i}P_{jt}+\mu_{it}$ | | | | | | |
| Blue portfolio | 0.0415*  (0.0070) | - | - | - | - | - |
| Purple portfolio | 0.5499*  (0.0078) | - | - | - | - | - |
| Green portfolio | 0.6154*  (0.0056) | - | - | - | - | - |
| Red portfolio | 0.7803*  (0.0018) | - | - | - | - | - |
| ***Model 2:*** $P_{it}=\delta_{1i}+ \theta_{1i}P_{jt}+ \theta_{2i}{S\&P500}_{it}+\mu_{it}$ | | | | | | |
| Blue portfolio | -0.0396*  (0.0066) | 0.8859*  (0.0152) | - | - | - | - |
| Purple portfolio | 0.5258*  (0.0084) | 0.1361*  (0.0174) | - | - | - | - |
| Green portfolio | 0.5184*  (0.0061) | 0.5643*  (0.0164) | - | - | - | - |
| Red portfolio | 0.7048*  (0.0020) | 0.3924*  (0.0052) | - | - | - | - |
| ***Model 3:*** $P_{it}=\delta_{1i}+ \theta_{1i}P_{jt}+ \theta_{2i}{S\&P500}_{it}+\theta_{3i}{Brent}_{it}+\mu_{it}$ | | | | | | |
| Blue portfolio | -0.0729*  (0.0061) | 0.8954*  (0.0140) | 0.2636*  (0.0050) | - | - | - |
| Purple portfolio | 0.4724*  (0.0087) | 0.1929*  (0.0173) | 0.1229*  (0.0061) | - | - | - |
| Green portfolio | 0.4560*  (0.0060) | 0.6143*  (0.0157) | 0.1883*  (0.0053) | - | - | - |
| Red portfolio | 0.6543*  (0.0021) | 0.4390*  (0.0051) | 0.1090*  (0.0017) | - | - | - |
| ***Model 4:*** $P_{it}=\delta_{1i}+ \theta_{1i}P_{jt}+ \theta_{2i}{S\&P500}_{it}{+ \theta}_{3i}{sentiments}_{it}+\mu_{it}$ | | | | | | |
| Blue portfolio | -0.0397  (.0066) | 0.8864*  (0.0153) | - | -0.0215  (0.0316) | - | - |
| Purple portfolio | 0.5248*  (0.0084) | 0.1394*  (0.0173) | - | -0.3466*  (0.0345) | - | - |
| Green portfolio | 0.5178*  (0.0061) | 0.5631*  (0.0165) | - | 0.1178*  (0.0310) | - | - |
| Red portfolio | 0.7041*  (0.0020) | 0.3925*  (0.0052) | - | 0.0505*  (0.0096) | - | - |
| ***Model 5:*** $P_{it}=\delta_{1i}+ \theta_{1i}P_{jt}+ \theta_{2i}GFC*{S\&P500}_{it}+\theta_{3i}NGFC*{S\&P500}_{it}{+\mu}_{it}$ | | | | | | |
| Blue portfolio | -0.0284*  (0.0066) | - | - | - | 0.1388*  (0.0033) | 0.0010*  (0.0000) |
| Purple portfolio | 0.5277*  (0.0084) | - | - | - | 0.0378*  (0.0036) | 0.0001*  (0.0000) |
| Green portfolio | 0.5234*  (0.0062) | - | - | - | 0.0005*  (0.0001) | 0.0004*  (0.0001) |
| Red portfolio | 0.7179*  (0.0020) | - | - | - | 0.0361*  (0.0001) | 0.0002*  (0.0000) |

Note: This table displays the long-term relationships depicted models (1-5). The dependent variable, $P_{it},$ is a panel of CEE stock markets. $P_{jt}$ is a S1 related portfolio of other sampled stock markets. $P_{jt}$ is a *S1* related portfolio of other sampled stock markets. Blue portfolio captures correlation of 0.2 or lower; purple portfolio with a correlation between 0.2 and 0.3; green portfolio with a correlation between 0.4 and 0.5; and red portfolio stock having a correlation of 0.6 or more. ${S\&P500}_{it}$ is the US market price index; ${Brent}_{it}$ is Brent oil price series; and ${senti}_{it}$ is global investor sentiment.${S\&P500}_{i}$ effects are examined during the GFC period (*GFC*) and the non-GFC period (*NGFC*) in model 5. * denotes significance at 5 percent or better.

**Table S7** Number of observations by portfolios and regions

| Portfolios | Number of observations |
| --- | --- |
| Yellow | 212844 |
| Blue | 13306 |
| Purple | 12885 |
| Green | 53219 |
| Red | 264015 |
| Yellow: Asia | 4435 |
| Blue: Asia | 168530 |
| Purple: Asia | 164096 |
| Green: Asia | 292710 |
| Red: Asia | 771681 |
| Yellow: Latin America | 70964 |
| Blue: Latin America | 124181 |
| Purple: Latin America | 48785 |
| Green: Latin America | 199575 |
| Red: Latin America | 532200 |
| Yellow: MENA | 186235 |
| Blue: MENA | 141920 |
| Purple: MENA | 159660 |
| Green: MENA | 226185 |
| Red: MENA | 736210 |
| Yellow: CEE | 124180 |
| Blue: CEE | 186270 |
| Purple: CEE | 124180 |
| Green: CEE | 150790 |
| Red: CEE | 984570 |
| Asia | 399151 |
| LA | 133051 |
| MENA | 26610 |
| CEE | 35480 |
